# Strongyle Infection and Gut Microbiota: Profiling of Resistant and Susceptible Horses Over a Grazing Season

**DOI:** 10.3389/fphys.2018.00272

**Published:** 2018-03-21

**Authors:** Allison Clark, Guillaume Sallé, Valentine Ballan, Fabrice Reigner, Annabelle Meynadier, Jacques Cortet, Christine Koch, Mickaël Riou, Alexandra Blanchard, Núria Mach

**Affiliations:** ^1^Department of Health Science, Open University of Catalonia, Barcelona, Spain; ^2^UMR 1282, Institut National de la Recherche Agronomique, Infectiologie et Santé Publique, Université François-Rabelais, Nouzilly, France; ^3^UMR 1313, Institut National de la Recherche Agronomique, AgroParisTech, Université Paris-Saclay, Jouy-en-Josas, France; ^4^UEPAO 1297, Institut National de la Recherche Agronomique, Unité Expérimentale de Physiologie Animale de l'Orfrasière, Nouzilly, France; ^5^UMR 1388, Institut National de la Recherche Agronomique, GenPhySE, Toulouse, France; ^6^UE-1277, Institut National de la Recherche Agronomique, Plate-Forme d'Infectiologie Expérimentale, Nouzilly, France; ^7^Pancosma SA, Geneva, Switzerland

**Keywords:** bacteria, cyathostomin, anaerobic fungi, gut microbiome, immunity, horse, protozoa

## Abstract

Gastrointestinal strongyles are a major threat to horses' health and welfare. Given that strongyles inhabit the same niche as the gut microbiota, they may interact with each other. These beneficial or detrimental interactions are unknown in horses and could partly explain contrasted susceptibility to infection between individuals. To address these questions, an experimental pasture trial with 20 worm-free female Welsh ponies (10 susceptible (S) and 10 resistant (R) to parasite infection) was implemented for 5 months. Fecal egg counts (FEC), hematological and biochemical data, body weight and gut microbiological composition were studied in each individual after 0, 24, 43, 92 and 132 grazing days. R and S ponies displayed divergent immunological profiles and slight differences in microbiological composition under worm-free conditions. After exposure to natural infection, the predicted R ponies exhibited lower FEC after 92 and 132 grazing days, and maintained higher levels of circulating monocytes and eosinophils, while lymphocytosis persisted in S ponies. Although the overall gut microbiota diversity and structure remained similar during the parasite infection between the two groups, S ponies exhibited a reduction of bacteria such as *Ruminococcus, Clostridium* XIVa and members of the *Lachnospiraceae* family, which may have promoted a disruption of mucosal homeostasis at day 92. In line with this hypothesis, an increase in pathobionts such as *Pseudomonas* and *Campylobacter* together with changes in several predicted immunological pathways, including pathogen sensing, lipid metabolism, and activation of signal transduction that are critical for the regulation of immune system and energy homeostasis were observed in S relative to R ponies. Moreover, S ponies displayed an increase in protozoan concentrations at day 92, suggesting that strongyles and protozoa may contribute to each other's success in the equine intestines. It could also be that S individuals favor the increase of these carbohydrate-degrading microorganisms to enhance the supply of nutrients needed to fight strongyle infection. Overall, this study provides a foundation to better understand the mechanisms that underpin the relationship between equines and natural strongyle infection. The profiling of horse immune response and gut microbiota should contribute to the development of novel biomarkers for strongyle infection.

## Introduction

Grazing horses are infected by a complex community of parasitic helminths, mainly strongyles (Ogbourne, 1976; Bucknell et al., 1995; Corning, 2009; Kuzmina et al., 2016). Like other worms, strongyles have a direct life cycle in which they can survive outside of its host in pastures as well as in the horse's intestines (Taylor et al., 2007). Infective larvae (L3 stage) are usually ingested and migrate to their preferred niche in the small or large intestine. After two molts, they will eventually become sexually mature adults and will lay eggs that are passed onto the pasture via feces (Taylor et al., 2007).

Equine strongyle species are classified into *Strongylinae* and *Cyathostominae*, which differ, among other criteria, by their respective size (Lichtenfels et al., 2008). *Strongylus vulgaris* is the most pathogenic of the large strongyles as a result of the intestinal infarction that larval stages can cause during their migration. *S. vulgaris* prevalence has been drastically reduced since the release of modern anthelmintics (Nielsen et al., 2012). In contrast, nearly all horses are infected by small strongyles or cyathostomins throughout the world (Ogbourne, 1976; Bucknell et al., 1995; Lyons et al., 1999). Small strongyles infections are responsible for milder symptoms, including weight loss or poor hair condition (Love et al., 1999). Infections are more common in young animals (Love et al., 1999) largely due to immunological hyporesponsiveness, meaning there is likely an immune component to infection susceptibility (Lyons et al., 1999). In fact, the prevalence of infections has been found to be lower in horses older than 5 years (Relf et al., 2013; Kornaś et al., 2015). However, larval stages encyst into the colonic mucosa as part of their life cycle and their massive emergence (larval cyathostominosis) can result in abdominal pain, diarrhea (Love et al., 1999; Matthews, 2014) and eventually death (Giles et al., 1985).

Drug inefficacy reports have accumulated worldwide over the recent years, and resistant cyathostomin isolates are now to be found in Europe (Geurden et al., 2014; Sallé et al., 2017), America (Smith et al., 2015), and Oceania (Scott et al., 2015). It is therefore required to alleviate drug selection pressure put on cyathostomin populations. Targeted-selective treatment can help reduce drug usage as horses differ in their intrinsic potential to resist infection. Indeed, it has been estimated that 80% of the total worm burden is produced by 20% of horses (Wood et al., 2012) and that 21% of the inter-individual variation has a heritable component (Kornaś et al., 2015). However, the factors underpinning this phenotypic contrast (Lester et al., 2013; Sallé et al., 2015) still remain unclear.

Equine strongyles are in close contact with a large community of microorganisms in the host intestines, estimated to reach a concentration of 10^9^ microorganisms per gram of ingesta in the cecum alone (Mackie and Wilkins, 1988), spanning 108 bacterial genera (Steelman et al., 2012; Mach et al., 2017; Venable et al., 2017) and at least seven phyla (Costa et al., 2012, 2015; Shepherd et al., 2012; Weese et al., 2015; Mach et al., 2017). Bacterial populations differ greatly throughout the various compartments of the equine gastrointestinal tract (e.g., duodenum, jejunum, ileum and colon) due to differences in the gut pH, available energy sources, epithelial architecture of each region, oxygen levels and physiological roles (Costa et al., 2015; Ericsson et al., 2016). The gut microbiota promotes digestion and nutrient absorption for host energy production and synthesizes folate (Sugahara et al., 2015), vitamin K_2_ (Marley et al., 1986) and short chain fatty acids (SCFA) such as acetate, butyrate and propionate (Nedjadi et al., 2014; Ericsson et al., 2016). The gut microbiota also protects the host from pathogens and stimulates and matures the immune system and epithelial cells (reviewed by Nicholson et al., 2012). Although bacteria represent the major portion of the hindgut microbiota in horses (Dougal et al., 2013; Fernandes et al., 2014), the quantity of anaerobic fungi, protozoa and archaea relative to total bacteria found in horse feces is about 0.26, 3.7, and 0.0015, respectively (Dougal et al., 2012).

The physical presence of helminths in the intestinal lumen can alter the gut microbiota activity and composition (Midha et al., 2017; Peachey et al., 2017). These perturbations have been studied in various host-nematode relationships including rodent models (Reynolds et al., 2014; Fricke et al., 2015; McKenney et al., 2015; Su et al., 2017), ruminants (Li et al., 2011, 2016; El-Ashram and Suo, 2017), pigs (Li et al., 2012; Wu et al., 2012), and cats (Duarte et al., 2016). However, it remains unresolved whether nematode infection has a beneficial (Lee et al., 2014) or a detrimental (Houlden et al., 2015) impact on the gut microbiota diversity, richness and functions. The mechanisms responsible for these gut microbiota alterations are also unclear and could arise indirectly due to the immune response helminths trigger in their host (Reynolds et al., 2014, 2015; Fricke et al., 2015; Cattadori et al., 2016; Zaiss and Harris, 2016), such as regulatory T cell stimulation and lymphoid tissue modifications and changes in the intestinal barrier (Boyett and Hsieh, 2014; Giacomin et al., 2016). On the other hand, the secretion of putative anti-bacterial compounds (Mcmurdie and Holmes, 2012; Holm et al., 2015) or modifications in the intestinal environment that supports helminth survival might directly modify the gut microbiota (D'Elia et al., 2009; Midha et al., 2017).

The putative interactions between equine strongyle infection, the gut microbiota and host physiology are unknown in horses. To address these questions, grazing ponies with an extreme phenotypic resistance or susceptibility toward natural strongyle infection were monitored over a 5-month period. We aimed to provide insights into the host response and the gut microbiological composition associated with strongyle natural infection in order to guide the development of new microbiota-based control strategies.

## Materials and methods

### Selection of animals

Twenty female Welsh ponies (10 resistant, R, and 10 susceptible, S; 5 ± 1.3 years old) were selected from an experimental herd of 107 ponies (INRA, UEPAO, Nouzilly, France) regularly monitored for fecal egg counts (FEC) during the grazing season since 2011. Selection was based on the FEC records database consisting of 753 observations (*n* = 146, 278, 214, and 115 for spring, summer, autumn, and winter measures) of the whole herd. Every individual pony had been recorded at least three times over a minimum of 2 years (seven observations per pony on average across the whole herd). FEC data were log-transformed to correct for over-dispersion and fitted a linear mixed model accounting for environmental fixed effects (month of sampling, year of sampling, time since last treatment, age at sampling). The individual was considered as a random variable to account for the intrinsic pony potential against strongyle infection. Estimated individual effects were centered and reduced to express each individual potential as a deviation from the mean on the logarithmic scale. Based on these estimates, the 20 individuals with most extreme estimates were chosen to create a resistant R group (mean individual effect with −1.18 phenotypic deviation from the mean and average age of 5.6 years) and a susceptible S group (mean individual effect with +1.45 phenotypic deviation from the mean and average age of 4.7 years). The median egg counts /g feces across the past 5 years were 800 for S (the mean was 897, the first quartile was 350, and the third quartile was 1,375), whereas the median egg counts/g feces were 0 for R (the mean was 24.6, the first quartile was 0, and the third quartile was 150; Figures S1A,B).

As it has been previously established that gut microbiota profiles might be shaped by host genetics (Lozupone et al., 2012; Goodrich et al., 2014), this information was considered to understand the individual variance underlying microbiota composition. The “kinship2” package in R was used to create the genetic relationship matrix. Both pedigree tree and correlation structure matrix are depicted in Figures S1C,D, respectively.

All the procedures were conducted according to the guidelines for the care and use of experimental animals established by the French Ministry of Teaching and Research and the regional Val de Loire Ethics Committee (CEEA VdL, n° 19). The protocol was registered under the number 2015021210238289_v4 in the experimental installations with the permit number: C371753. All the protocols were conducted in accordance with EEC regulation (n° 2010/63/UE) governing the care and use of laboratory animals and effective in France since the 1st of January 2013.

### Animal sampling

The 20 Welsh ponies were treated with moxidectin and praziquantel (Equest Pramox®, Zoetis, Paris, France, 400 μg/kg of body weight of moxidectin and 2,5 mg/kg of praziquantel) in March 2015 to clear any patent and pre-patent infection and kept indoors for 3 months until the end of the moxidectin residual period. They were maintained under natural light conditions in a 240 m^2^ pen with slatted floors, which precluded further nematode infections until they were moved to the experimental pasture. During housing, animals were fed with hay *ad libitum* and 600 g concentrate per animal per day. The concentrate (Tellus Thivat Nutrition Animale Propriétaire, Saint Germain de Salles, France) consisted of barley (150 g/kg), oat bran (162 g/kg), wheat straw (184.7 g/kg), oats (200 g/kg), alfalfa (121.7 g/kg), sugar beet pulp (50 g/kg), molasses (30 g/kg), salt (7.3 g/kg), carbonate Ca (5.5 g/kg), and a mineral and vitamin mix (2 g/kg), on an as-fed basis. The mineral and vitamin mix contained Ca (28.5%), P (1.6%), Na (5.6%), vitamin A (500,000 IU), vitamin D_3_ (125,000 IU), vitamin E (1,500 IU), cobalt carbonate (42 mg/kg), cupric sulfate (500 mg/kg), calcium iodate (10 mg/kg), iron sulfate (1,000 mg/kg), manganese sulfate (5,800 mg/kg), sodium selenite (16 mg/kg), and zinc sulfate (7,500 mg/kg) on an as-fed basis.

Grazing started by the end of a 3-month moxidectin residual period (mid-June 2015). Ponies grazed from mid-June to the end of October 2015 at the Nouzilly experimental station (France). The experimental pasture (7.44 ha) consisted of *Festuca arundinacea, Phleum prateonse, Poa abbreviate, Holcus lanatus*, and *Dactylis glomerata*. During all phases of the experimental period, ponies were provided access to water *ad libitum*.

Moxidectin is known to have moderate efficacy against encysted cyathostomin larvae (Xiao et al., 1994; Reinemeyer et al., 2015). Therefore, egg excretion can occur at the end of the residual period, as a result of encysted larvae completing their development into adults. To eliminate this residual excretion and to avoid any interference with strongyle infection at pasture, a treatment targeting the only luminal immature and adult stages (Strongid® paste, Zoetis, Paris, France; single oral dose of 1.36 mg pyrantel base per kg of body weight) was implemented at day 30.

**Figure 1:**
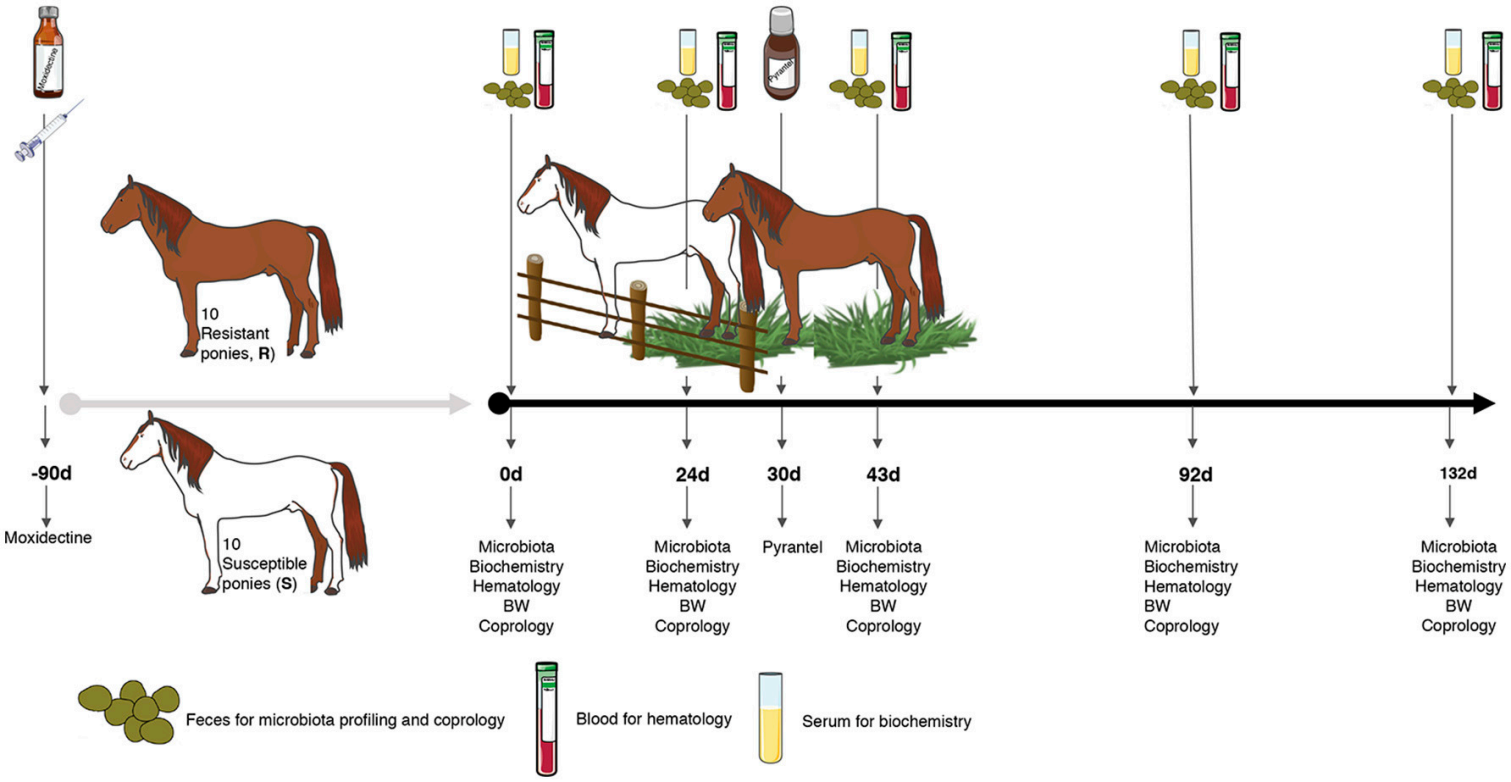
Experimental design and sampling. A set of 20 female ponies [10 susceptible (S) and 10 resistant (R) to strongylosis] were selected based on their fecal egg counts history during previous pasture seasons and were kept inside during the winter. In the spring, they were treated with moxidectin, to ensure that they were totally free from gastrointestinal nematodes (even from putative encysted larvae) and were kept indoors for 3 months. Thereafter, once the effect of moxidectin treatment was no longer detected, the ponies were moved to a 7.44 ha pasture to start the study. At day 30 of the study, a pyrantel treatment was administered to all the animals in order to eliminate any residual infections that could interfere in the protocol. A longitudinal monitoring of the parasitism level in each animal was performed through five time points from June to October. At each time point, fecal samples were collected from all ponies on 0, 24, 43, 92, and 132 days after the beginning of the grazing season to carry out fecal egg counts, pH measurements, and microbiota profiling. Blood samples were taken at the same time points to analyze biochemical and hematological parameters. Body weight (BW) was also recorded at the same time points.

Every pony was subjected to a longitudinal monitoring of fecal strongyle egg excretion, feces microbiota, blood biochemistry, hematology and body weight, e.g., 0, 24, 43, 92, and 132 days after the onset of grazing (Figure 1).

Fecal samples were collected from the rectum. Fecal aliquots for microbiota analysis were immediately snap-frozen in liquid nitrogen and stored at −80°C until DNA extraction, whereas fecal aliquots to measure the fecal egg counts were immediately sent to the laboratory.

The pH in the feces was determined after 10% fecal suspension (wt/vol) in saline solution.

Blood samples were taken from each pony and collected in EDTA-K3-coated tubes (5 mL) to determine hematological parameters and heparin tubes (10 mL) to determine biochemical parameters. After clotting, the heparin tubes were centrifuged at 4,000 rpm during 15 min and the harvested plasma was stored at −20°C until analysis. Additionally, blood collected in EDTA-K3-tubes was used to measure the different blood cells.

For each individual, body weight was measured monthly and average daily weight gain was also calculated.

None of the ponies received antibiotic therapy during the sampling period and diarrhea was not detected in any ponies.

### Fecal egg counts

Fecal egg counts (eggs per gram of wet feces) was measured as a proxy for patent strongyle infection. FEC was carried out using a modified McMaster technique (Raynaud, 1970) on 5 g of feces diluted in 70 mL of NaCl solution with a density of 1.2 (sensitivity of 50 eggs/g). A Wilcoxon rank-sum test with Benjamini-Hochberg multiple test correction was used to determine whether there was a significant difference between groups across the experiment. An adjusted *p* < 0.05 was considered significant.

### Blood hematological and biochemical assays

For blood hematological assays, blood was stirred for 15 min at room temperature to facilitate oxygenation. Different blood cells were analyzed, including leucocytes (lymphocytes, monocytes, neutrophils, basophils and eosinophils), erythrocytes and different blood parameters such as hematocrit, mean corpuscular volume and the thrombocytes. The total blood cells were counted with a MS9-5 Hematology Counter® (digital automatic hematology analyzer, Melet Schloesing Laboratories, France).

The serum biochemical parameters (albumin, cholesterol, globin, glucose, phosphatase alkaline, total proteins and urea) were measured with a colorimetric method using Select-6V rings with the M-Scan II Biochemical analyzer (Melet Schloesing Laboratories, France).

Mixed-effects analysis of the variance (ANOVA) or Wilcoxon rank-sum tests were conducted for continuous variables fitting a normal or non-normal distribution respectively to delineate whether there was a significant difference between the average values of phenotype traits for the different groups, using a significance level of *p* < 0.05. Blood cell counts were corrected for the mild dehydration by fitting the hematocrit as a co-variable in the model.

### Weather data

Daily precipitation and temperatures were recorded at a meteorological station located 14 km from the experimental area.

### Pasture contamination analysis

Pasture contamination, expressed as the number of infective larvae (L3) per kg of dry herbage, was measured throughout the grazing season. At every occasion, four plucked grass were picked up in 100 random locations across pasture (Gruner and Raynaud, 1980). A sub-sampling of 600 g of collected grass was mixed with 20 mL of neutral pH soap diluted in 5 L of tap water and left at room temperature for 24 h. Grass was then separated using a gross sieve (0.5 cm mesh) and discarded. The 5 L water was then passed through both a 125 μm mesh sieve and a 20 μm mesh sieve to retain smaller particles, including strongyle larvae. Recovered particles were diluted in 40 mL of tap water and subsequently dispatched into 4 × 10 mL glass tubes. A flotation technique was used to isolate larvae from other abiotic contaminants as follows. Tubes were centrifuged at 2,500 rpm for 5 min, before discarding supernatant and adding a dense solution (NaCl, density 1.18–1.2) and a cover slip onto glass tube. Gentle centrifuge was subsequently performed at 1,500 rpm for 8 min thus allowing larval material adherence onto the cover slip that was further examined under an optical microscope. This last step was performed three times.

### Forage chemical analysis

Hay was sampled at day 0 via grab samples from multiple depths into a bale and then composited. Herbage samples were collected from three randomly selected zones in the experimental pasture at days 24, 43, 92, and 132. At each sampling time, 10 hand-plucked samples simulating ≪ bites ≫ were taken from pasture. All samples were frozen until chemical analyses. Chemical compositions of samples were determined according to the “Association Française de Normalisation” (AFNOR) procedures: NF V18-109 (AFNOR, 1982) for dry matter (DM); NF V18-101 (AFNOR, 1977b) for crude ash; NF V18-100 (AFNOR, 1977a) for crude proteins; NF V03-040 (AFNOR, 1993) for crude fiber; NF V18-117 (AFNOR, 1977a) for crude fat; NF V18-122 (AFNOR, 1997) for neutral detergent fiber (NDF), acid detergent fiber (ADF), and acid detergent lignin (ADL). All of them were assayed with a heat stable amylase and expressed with exclusive of residual ash according to the method of Van Soest et al. (1991). Non-fiber carbohydrate (NFC), also called neutral detergent soluble carbohydrate (NDCS) were obtained by calculations: NFC = 1,000–CP–CF–Mm–NDF.

### Microorganisms DNA extraction from feces samples

Total DNA was extracted from aliquots of frozen fecal samples (200 mg; 100 samples at different time points from 20 ponies), using E.Z.N.A.® Stool DNA Kit (Omega Bio-Tek, Norcross, Georgia, USA). The DNA extraction protocol was carried out according to the manufacturer's instructions (Omega- Bio-Tek, Norcross, Georgia, USA).

### V3-V4 16S rRNA gene amplification

The V3-V4 hyper-variable regions of the 16S rDNA gene were amplified with two rounds of PCR using the forward primer (5′-CTTTCCCTACACGACGCTCTTCCGATCTACGGRAGGCAGCAG-3′) and the reverse primer (5′-GGAGTTCAGACGTGTGCTCTTCCGATCTTACCAGGGTATCTAATCCT-3′) modified in order to include Illumina adapters and barcode sequences which allow for directional sequencing. The targeted region resulted in an amplicon of a region of ~459 bp that when sequenced with paired-end reads had at least ~50 bp of overlapping sequence in the middle. The first round of amplification was performed in triplicate in a total volume of 50 μL containing 10 ng of DNA, 2.5 units of a DNA-free Taq DNA Polymerase and 10X Taq DNA polymerase buffer (MTP Taq DNA Polymerase, Sigma). Subsequently, 10 mM of dNTP mixture (Euromedex, Souffelweyersheim, France), 20 mM of each primer (Sigma, Lezennes, France) and Nuclease-free water (Ambion, Thermo Fisher Scientific, Waltham, USA) were added. Ultrapure Taq DNA polymerase, ultrapure reagents, and plastic were selected in order to be DNA-free. The thermal cycle consisted of an initial denaturation step (1 min at 94°C), followed by 30 cycles of denaturation (1 min at 94°C), annealing (1 min at 65°C) and 1 min of extension at 72°C. The final extension step was performed for 10 min at 72°C. Amplicons were then purified using magnetic beads (Clean PCR system, CleanNA, Alphen an den Rijn, The Netherlands) as follows: beads/PCR reactional volume ratio of 0.8X and final elution volume of 32 μL using Elution Buffer EB (Qiagen). The concentrations of the purified amplicons were checked using a NanoDrop 8000 spectrophotometer (Thermo Fisher Scientific, Waltham, USA).

Sample multiplexing was performed using 6 bp unique indexes, which were added during the second PCR step at the same time as the second part of the P5/P7 adapters used for the sequencing step on the Illumina MiSeq flow cells with the forward primer (5′-AATGATACGGCGACCACCGAGATCTACACTCTTTCCCTACACGAC-3′) and reverse primer (5′-CAAGCAGAAGACGGCATACGAGATNNNNNNGTGACTGGAGTTCAGACGTGT-3′).

This second PCR step was performed using 10 ng of purified amplicons from the first PCR and adding 2.5 units of a DNA-free Taq DNA Polymerase and 10X MTP TaqDNA polymerase buffer (Sigma). The buffer was complemented with 10 mM of dNTP mixture (Euromedex), 20 mM of each primer (Eurogentec, HPLC grade) and Nuclease-free water (Ambion, Life Technologies) up to a final volume of 50 μL. The PCR reaction was carried out as follows: an initial denaturation step (94°C for 1 min), 12 cycles of amplification (94°C for 1 min, 65°C for 1 min and 72°C for 1 min) and a final extension step at 72°C for 10 min. Amplicons were purified as described for the first PCR round. The concentration of the purified amplicons was measured using Nanodrop 8,000 spectrophotometer (Thermo Scientific) and the quality of a set of amplicons (12 samples per sequencing run) was checked using DNA 7,500 chips onto a Bioanalyzer 2,100 (Agilent Technologies, Santa Clara, CA, USA). All libraries were pooled at equimolar concentration in order to generate equivalent number of raw reads with each library. The final pool had a diluted concentration of 5 nM to 20 nM and was used for sequencing. Amplicon libraries were mixed with 15% PhiX control according to the Illumina's protocol. Details on sequencing, PhiX control and FastQ files generation are specified elsewhere (Lluch et al., 2015). For this study, one sequencing run was performed using MiSeq 500 cycle reagent kit v2 (2 × 250 output; Illumina, USA).

### Sequencing data preprocessing

Sequences were processed using the version 1.9.0 of the Quantitative Insights Into Microbial Ecology (QIIME) pipeline (Caporaso et al., 2010; Rideout et al., 2014) and by choosing the open-reference operational taxonomic units (OTU) calling approach (Rideout et al., 2014).

First, forward and reverse paired-end sequence reads were collapsed into a single continuous sequence according to the “fastq-join” option of the “join_paired_ends.py” command in QIIME. The fastq-join function allowed a maximum difference within overlap region of 8%, a minimum overlap setting of 6 bp and a maximum overlap setting of 60 bp. The reads that did not overlap (~20% of the total) were removed from the analysis. The retained sequences were then quality filtered. De-multiplexing, primer removal and quality filtering processes were performed using the “split_libraries”_fastq.py command in QIIME. We applied a default base call Phred threshold of 20, allowing maximum three low-quality base calls before truncating a read, including only reads with >75% consecutive high-quality base calls, and excluding reads with ambiguous (N) base calls (Navas-Molina et al., 2013).

Subsequently, the sequences were clustered into OTUs against the GreenGenes database (release 2013-08: gg_13_8_otus) (DeSantis et al., 2006) by using the uclust (Edgar, 2010) method at a 97% similarity cutoff. The filtering of chimeric OTUs was performed by using Usearch version 6.1 (Edgar et al., 2011) against the GreenGenes reference alignment (DeSantis et al., 2006). A phylogenic tree was generated from the filtered alignment using FastTree (Price et al., 2010). Singletons were discarded from the dataset to minimize the effect of spurious, low abundance sequences using the “filter_otus_from_otu_table.py” script in QIIME. To confirm the annotation, the resulting OTU representative sequences were then searched against the Ribosomal Database Project naïve Bayesian classifier (RDP 10 database, version 6; Cole et al., 2009) database, using the online program SEQMATCH (http://rdp.cme.msu.edu/seqmatch/seqmatch_intro.jsp). Finally, consensus taxonomy was provided for each OTU based on the taxonomic assignment of individual reads using GreenGenes and RDP databases. Using OTU abundance and the corresponding taxonomic classifications, feature abundance matrices were calculated at different taxonomic levels, representing OTUs and taxa abundance per sample. The “Phyloseq” (Mcmurdie and Holmes, 2012) and “Vegan” (Dixon, 2003) R package were used for the detailed downstream analysis on abundance matrix.

In the end, a total of 8,010,052 paired-end 250 bp reads were obtained, 6,428,315 of which were retained as high-quality sequences (Table S1). On average, a total of 58,015 sequences per sample were obtained in the study, with a mean length of 441 ± 15 bp. These sequences were clustered into 15,784 OTUs using the open reference-based OTU-picking process (Table S2). Among them, 12,069 were classified taxonomically down to the genus level (Table S2). OTU counts per sample and OTU taxonomical assignments are available in Table S2. The unclassified taxa were filtered out from the analysis because the main goal of the current study was to identify specific taxa related to host susceptibility to strongyle infection.

The α-diversity indexes (observed species richness, Chao, 1984 and Shannon, 1997) were calculated using the “Phyloseq” R package (Mcmurdie and Holmes, 2012). Shannon's diversity index is a composite measure of richness (number of OTUs present) and evenness (relative abundance of OTUs). The non-parametric Kruskal-Wallis test was used to compare α-diversity indexes between groups and between the different time points. If the Kruskal–Wallis test was significant between the different time points, the *post-hoc* Dunn test analysis, which was performed with the *dunnTest* function in the “FSA” package, was applied to determine which levels of the independent variable differ from each other level.

Relative abundance normalization was applied, which divides raw counts from a particular sample by the total number of reads in each sample.

To estimate β-diversity, un-weighted and weighted UniFrac distances were calculated from the OTU abundance tables, and used in principal coordinates analysis (PCoA), correspondence analysis (CA), and non-parametric multidimensional scaling (NMDS) with the “Phyloseq” R package. The Permutational Multivariate Analysis of Variance (PERMANOVA) on un-weighted and weighted UniFrac distance matrices were applied through the *adonis2* function with 9,999 permutations from “Vegan” R package to test for groups effect. Moreover, Bray-Curtis similarity coefficients were calculated from genera count matrix and plotted in a NMDS and CA graph to show the similarity among samples using the “Vegan” R package. Then after, the PERMANOVA test was also applied to test for groups' and time points' effect.

In addition to multivariate analysis, we used the analysis of similarities (ANOSIM) to test for intragroup dispersion. ANOSIM is a permutation-based test where the null hypothesis states that within-group distances are not significantly smaller than between-group distances. The test statistic (*R*) can range from 1 to −1, with a value of 1 indicating that all samples within groups are more similar to each other than to any other samples from different groups. *R* is ≈0 when the null hypothesis is true, that distances within and between groups are the same on average. Because multiple comparison corrections for ANOSIM were not available, the number of permutations being used on those calculations was increased to 9,999.

The Wilcoxon rank-sum test with Benjamini-Hochberg multiple test correction was used to determine the differentially abundant OTUs, phyla, families, and genera between groups. An adjusted *p* ≤ 0.25 was considered significant. This threshold was employed in previous microbiome studies because allows compensation for the large number of microbial taxa and multiple comparison adjustment (Lim et al., 2017).

This targeted locus study project has been deposited at DDBJ/EMBL/GenBank under the accession KBTQ01000000. The version described in this paper is the first version, KBTQ01000000. The bioproject described in this paper belongs to the BioProject PRJNA413884. The corresponding BioSamples accession numbers were SAMN07773451 to SAMN07773550.

### Functional metagenomic predictions

The functional prediction for the 16S rRNA marker gene sequences was done using the phylogenetic investigation of communities by reconstruction of unobserved states.

(PICRUSt) (Langille et al., 2013). After excluding the unknown OTUs from the GreenGenes reference database and normalizing by 16S rRNA gene copy number, functional metagenomes for each sample were predicted from the Kyoto Encyclopedia of Genes and Genomes (KEGG) catalog and collapsed to a specified KEGG level. We used Wilcoxon rank-sum test with Benjamini-Hochberg multiple test correction to evaluate predicted pathway-level enrichments between groups. An adjusted *p* < 0.05 was considered as significant.

### Network inference at the genus level

Networks at the genus level were inferred between groups at different time points. In order to prevent the compositional effects bias typical of the classical correlations methods, we calculated the correlations among genera using the partial correlation and information theory (PCIT) approach, which identifies significant co-occurrence patterns through a data-driven methodology based on partial correlation and information theory as implemented in the PCIT algorithm (Reverter and Chan, 2008). Further details are depicted in Ramayo-Caldas et al. (2016). The genera with <0.1% mean relative abundances were excluded to acquire the results for the taxa that met the statistical conditions for correlation estimations. Nodes in the network represent the genera and edges that connect these nodes represent correlations between genera. Based on correlation coefficient and *p*-values for correlation, we constructed co-occurrence networks. The cutoff of *p*-values was 0.05. The cutoff of correlation coefficients was determined as *r* ≥ |0.35|. Network properties were calculated with the NetworkAnalyzer plugin in Cytoscape. We used the “iGraph” package in R to visualize the network. Strong and significant correlation between nodes (*r* ≥ |0.60|) were represented with larger edge width in the network.

### Real-time quantitative PCR (qPCR) analysis of bacterial, anaerobic fungal, and protozoan concentrations

Concentrations of protozoa, anaerobic fungi and bacteria in fecal samples were quantified using a QuantStudio 12K Flex real-time instrument (Thermo Fisher Scientific, Waltham, USA). Primers for real-time amplification of protozoa (FOR: 5′-GCTTTCGWTGGTAGTGTATT-3′; REV: 5′-CTTGCCCTCYAATCGTWCT-3′), anaerobic fungi (FOR: 5′-TCCTACCCTTTGTGAATTTG-3′; REV: 5′-CTGCGTTCTTCATCGTTGCG-3′) and bacteria (5′-CAGCMGCCGCGGTAANWC-3′; REV: 5′-CCGTCAATTCMTTTRAGTTT-3′) have already been described in Mach et al. (2017). Primers have been purchased from Eurofins Genomics (Ebersberg, Germany).

Amplified fragments of the target genes were used and diluted 10-fold in series to produce seven standards, ranging from 2.25 × 10^7^ to 2.25 × 10^13^ copies per μg of DNA for bacteria and protozoa and ranging from 3.70 × 10^6^ to 3.70 × 10^12^ copies per μg of DNA for anaerobic fungi. Each reaction contained, in a final volume of 20 μL, 10 μL of Sybergreen Mix (Power SYBR Green PCR Master Mix, ThermoFisher, Ullkirch-Graffenstaden, France), 0.6 μM of each primer to final concentration of 300 mM, and 2 μL of standard or DNA template at 0.5 ng/μL. The primer concentration of anaerobic fungi was 200 mM and 150 mM for protozoa. The DNA template was 0.5 ng/μL. In all cases, the thermal protocol for qPCR amplification and detection included an initial step of denaturation of 10 min (95°C), followed by 40 amplification cycles (15 s at 95°C; 60 s at 60°C). After each run, melting curves between 60 and 95°C were evaluated to confirm the absence of nonspecific signals. For each sample and each gene, qPCR runs were performed in triplicate. The standard curve obtained from the reference genomic fragment was used to calculate the number of copies of bacteria, protozoa or anaerobic fungi in feces. Taking into account the molecular mass of nucleotides and fragment length, we calculated the copy number as follows:

Copy number per nanogram = (NL ^*^ A ^*^ 10^−9^)/ (*n* ^*^ *mw*), where NL is the Avogadro constant (6.02 × 10^23^ molecules per mol), A was the molecular weight of DNA molecules (ng), *n* is the length of the amplicon in base pairs or nucleotides, and *mw* is the molecular weight per bp or nucleotide.

Wilcoxon rank-sum tests were calculated for all possible group combinations. A *p* < 0.05 was considered significant.

### Figure edition

Figures 1, **6** were produced using Servier Medical Art, available from https://smart.servier.com/.

## Results

The effects of natural strongyle infection on gut microbiota composition and host phenotypic variables were determined in ten resistant and ten susceptible grazing ponies over a 5-month grazing season (Figure 1). Microbiological, parasitological, hematological and biochemical measures were performed at five time points (Figure S2), hereafter referred to as days after the onset of the grazing season or grazing days (gd).

### A mixture of abiotic and biotic environmental stressors during the 43-day transitioning period induced shifts in immunological and microbiological profiles

**Figure 2:**
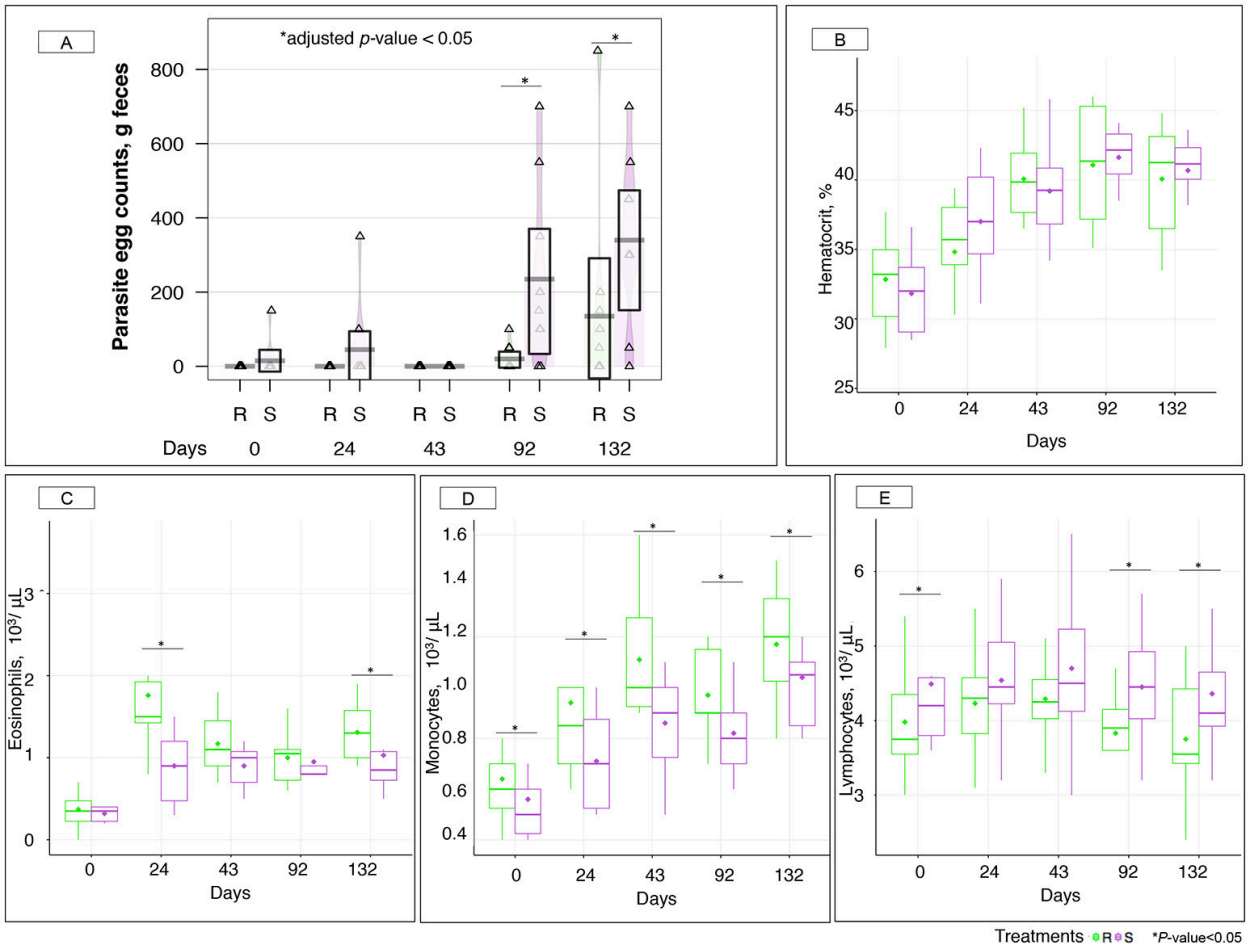
Parasite fecal egg counts and hematological parameters between susceptible and resistant animals across time. **(A)** Boxplot of the log parasite fecal egg counts (eggs/g feces) in susceptible (S) and resistant (R) animals. Purple and green stand for S and R ponies respectively. ^*^, adjusted *p* < 0.05 for comparison between S and R ponies in each time point; **(B)** Hematocrit (%) between S (purple boxes) and R (green boxes) animals across time. The quantification of different type of leukocytes: eosinophils **(C)**; monocytes **(D)**; and lymphocytes **(E)** were described between susceptible (S, purple boxes) and resistant (R, green boxes) animals across time. In all cases, boxes show median and interquartile range, and whiskers indicate 5th to 95th percentile. ^*^p < 0.05 for comparison between S and R ponies in each time point. Each triangle represents the parasite egg counts of each animal.

Measured FEC demonstrated a residual egg excretion in one susceptible individual at day 0 and in two susceptible ponies after 24 gd (Figure 2A). This was due to the known imperfect efficacy of moxidectin against encysted stages of strongyle. Because we were interested in studying the effects of strongyle exposure on the gut microbiota, a pyrantel treatment was administered after 30 gd to reset luminal parasite stages to zero in every pony. This treatment had a short-lived effect and resulted in zero FECs at 43 gd (Figure 2A). Therefore, the 0–43 gd period was considered as a transitioning period resulting in mild parasite exposure and changes in diet composition, management, and environmental conditions.

Indeed, ponies shifted from an indoor lifestyle (associated with a hay-concentrate based diet) to an outdoor pasture-based diet at day 0. Additionally, a heat wave took place over the first 43 gd resulting in almost no rainfall (0.2 mm) and high temperatures peaking at 37.7°C (Figure S3A). This situation ultimately led to grass senescence and lowered pasture quality (Figure S3B). Mild dehydration also occurred in ponies as supported by a constant rise in measured hematocrits from 32.33 to 39.63% during the first 43 gd (Figure 2B). To account for this, blood cell counts were corrected by hematocrit through time. Recorded hematological data showed that ponies were neither anemic nor thrombocytopenic throughout the experiment (Figure S4).

Concomitantly to these challenging conditions, increased levels of some white blood cell populations were observed, namely eosinophils (2.75-fold increase, *p* = 5.98 × 10^−13^, Figure 2C), and monocytes (1.64-fold increase, *p* = 2.94 × 10^−7^; Figure 2D) during the first 43 gd. Notably, R ponies had significantly higher levels of these immunological cells relative to S ponies (Figures 2C,D), while S ponies presented higher levels of lymphocytes (Figure 2E). Grass samples analysis did not provide any evidence of infective larvae until 43 gd.

**Figure 3:**
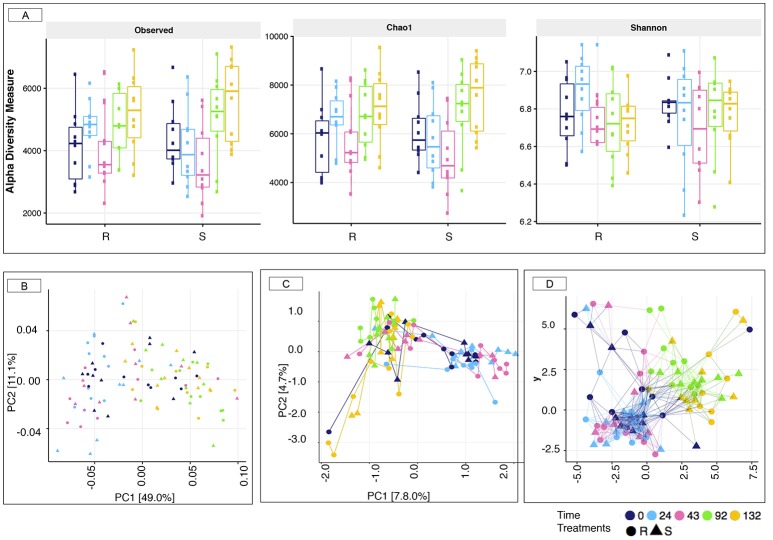
Estimation of the α-diversity indexes and β-diversity in susceptible and resistant animals across the experiment. **(A)** Estimation of the α-diversity indexes in susceptible (S) and resistant (R) across the experiment. The box color indicates the time point analyzed: (dark blue = 0 days, cyan = 24 days, pink = 43 days, green = 92 days, and yellow = 132 days); **(B)** Principal Coordinate analysis of weighted Unifrac distances to compare fecal communities at the level of OTUs that differ between S and R animals across the experiment. Both PC axes 1 and 2 were plotted. Together they explained 60.1% of whole variation; **(C)** Correspondence analyses of weighted Unifrac distances to compare fecal communities at the level of OTUs that differ between S and R animals across natural parasite infection. Both CA axes 1 and 2 were plotted; **(D)** Genus-level network representation between ponies across the experiment linked within a specified Jaccard distance of 0.85. Two samples were considered “connected” if the distance between them was <0.85. In all cases, the relative position of points was optimized for the visual display of network properties. The point's shape indicates the susceptibility to strongylosis (triangle: S; round: R), the node color indicates the time point analyzed: (dark blue = 0 days, cyan = 24 days, pink = 43 days, green = 92 days, and yellow = 132 days).

16S rRNA gene sequencing was used to profile the fecal microbiota of S and R ponies through time. In spite of contrasted susceptibility to parasite infection, measures of α-diversity (Observed species, Chao1 richness, and Shannon diversity index) were not significantly different between the two groups (Wilcoxon rank-sum test, *p* > 0.05, Figure 3A). Furthermore, the overall community structure showed no statistically significance difference in un-weighted (presence/absence) Unifrac analysis (PERMANOVA, *p* > 0.05) or abundance-weighted analysis (PERMANOVA, *p* > 0.05) during the 43 days transitioning period at the OTUs level (data not shown). Nevertheless, a large variation on the β-diversity was observed at 43 gd (Figure 3), with half of the individuals (4 S and 6 R ponies) clustering separately from all the other samples (ANOSIM test, *R* = 0.289, *p* < 0.001). This stratification matched with the age of animals. The gut microbiota species composition and spatial organization at day 43 in the oldest animals (4 S and 6 R, 6 ± 0.81 years old) were similar to that of day 0 and 24, whereas the gut microbiota of the youngest animals at 43 gd (4 R and 6 S, 4.3 ± 1.15 years old) was comparable to that of day 92 and 132 gd.

**Figure 4:**
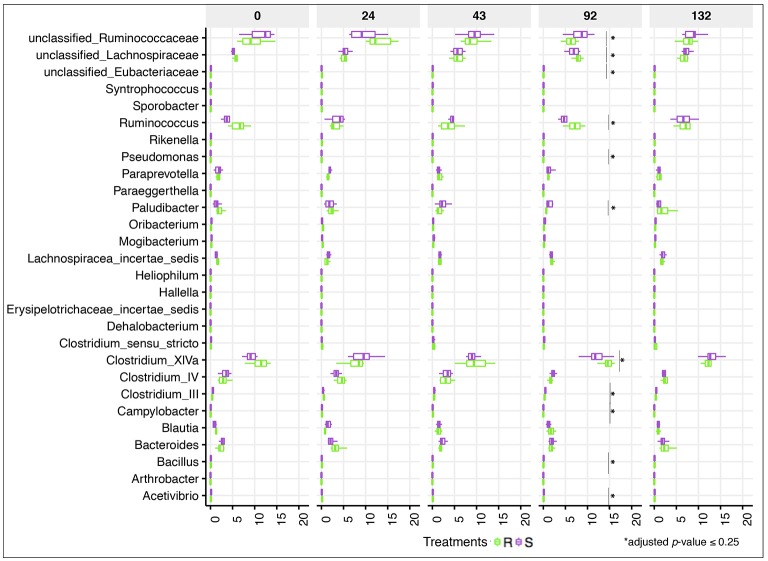
Dynamics of gut bacterial genera between susceptible and resistant animals across time. Boxplot graph representation of genera significantly affected between susceptible (S) and resistant (R) animals across time. In all cases, S animals are colored in purple and R animals in green. Boxes show median and interquartile range, and whiskers indicate 5th to 95th percentile. ^*^Adjusted *p* ≤ 0.25 for comparison between S and R ponies in each time point. Bacterial genera without ^*^are significant at a nominal *p* < 0.05.

**Figure 5:**
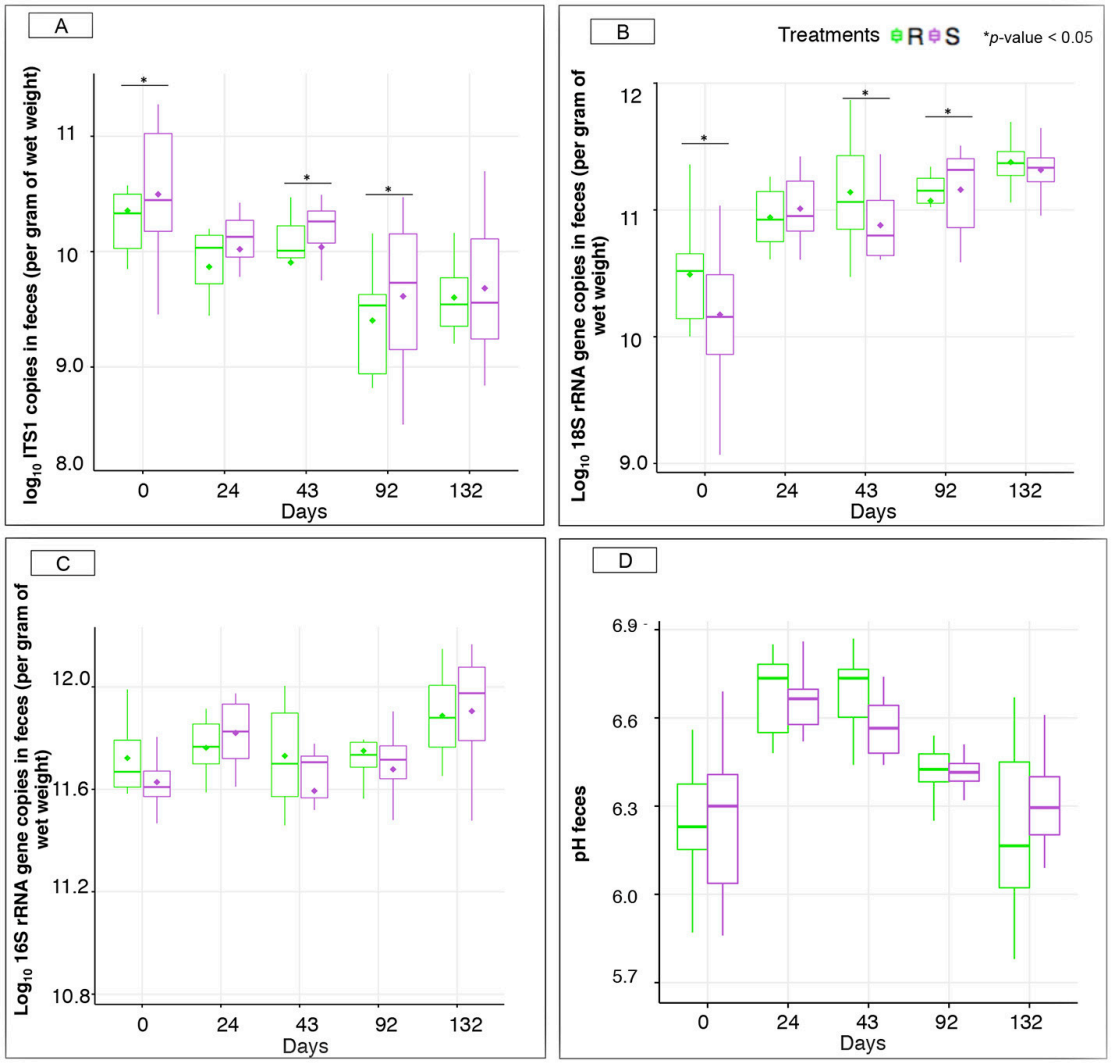
Microbiological concentrations and pH between susceptible and resistant ponies across time. **(A)** Boxplot graph representation of concentrations of anaerobic fungi in feces between S and R animals at different time points; **(B)** Boxplot graph representation of concentrations of protozoa in feces between S and R animals at different time points; **(C)** Boxplot graph representation of concentrations of bacteria in feces between S and R animals at different time points; **(D)** Boxplot graph representation of pH in feces between susceptible (S) and resistant (R) animals at different time points. In all cases, S animals are colored in purple and R animals in green. ^*^p < 0.05 for comparison between S and R ponies in each time point.

Despite the lack of significant alterations in the overall gut microbial community structure between S and R ponies during the 43 gd, we next evaluated the association between the abundance of specific gut microbial taxa and the susceptibility to parasite infection. No significant differences were identified at the phylum or family levels (data not shown), nor at the genera level (Figure 4, Table S3). Firmicutes (49.9 ± 5.30%), Bacteroidetes (34.5 ± 7.03%), Fibrobacteres (6.4 ± 3.76%) and Spirochaetes (6.3 ± 2.67%) phyla accounted for most of gut microbiota structure, while *Ruminococcaceae* (21.7 ± 4.02%), *Lachnospiraceae* (20.3 ± 4.66%), *Prevotellaceae* (8.7 ± 3.18%), *Porphyromonadaceae* (8.2 ± 2.37%), *Fibrobacteraceae* (6.5 ± 3.83%), and *Spirochaetacea* (6.4 ± 2.71%) were the most abundant families across samples. At the genus level, *Clostridium* XIVa (10.9 ± 3.00%) outranked members of the unclassified *Ruminococcaceae* family (9.4 ± 3.05%), *Fibrobacter* (7.6 ± 4.28%), *Treponema* (7.4 ± 3.03%), and *Prevotella* (6.7 ± 2.68%). The 20 most abundant bacterial genera for each group are shown in Figure S5. Predicted pathway analysis showed no significantly different biochemical pathways between the two groups during the first 43 gd (Table S4). The only observed differences between S and R during the first 43 gd of the trial was a higher anaerobic fungal concentrations (Figure 5A) and lower protozoan concentrations (Figure 5B) in S relative to R, whereas the bacterial concentrations (Figure 5C) and pH (Figure 5D) remained similar between groups of ponies within each time point.

### The predicted levels of resistance matched observed fecal egg counts between resistant and susceptible ponies and shifts in the gut microbiological composition

By the end of the transition period, e.g., after 43 gd, ponies were considered adapted to the diet and their new environmental conditions. Following the patent infection, parasite egg excretion was significantly higher (adjusted *p* < 0.05) in the S group after 92 (235 eggs in S and 20 eggs in R ponies on average) and 132 gd (340 eggs in S and 135 eggs in R ponies on average; Figure 2A).

Ponies' body weights (Figure S6A) and average daily weight gains (Figure S6B) did not show significant differences between groups after natural parasite infection, and none of them displayed clinical symptoms like lethargy or diarrhea. However, strongyle exposure induced an increase of circulating monocyte levels, which were higher (*p* < 0.05) in R in comparison to S ponies (Figure 2D). But the opposite trend was found for circulating lymphocytes, which continued to be significantly enriched in the white blood cells population in S ponies during parasite infection (Figure 2E). Among the white blood cell population, R ponies also presented higher levels of eosinophils at 132 gd (*p* < 0.05, Figure 2C). Serum biochemical analyses revealed that the enzyme alkaline phosphatase continued to be significantly higher (*p* < 0.05) in R than S during the patent parasite infection, whereas the levels of albumin, cholesterol, globins, glucose, total proteins and urea remained similar between both groups (Figure S7).

Strongyle mediated alterations in microbiota diversity and structure were investigated between the two groups of ponies. As for the transition period, both groups of ponies displayed equivalent microbial species richness and α-diversity indexes (*p* > 0.05, Figure 3A). However, a substantial increase in observed species richness and Chao1 indexes occurred at 92 and 132 gd relative to the other time points (*p* < 0.05; Dunn test; Figure S8). The weighted UniFrac distance followed by PCoA on the abundance table of OTUs (Figure 3B) showed no distinct clustering between samples from the S and the R group, which was indicative of, if at all, minor differences in microbiota composition between the two groups of ponies during parasite infection. Nonetheless, the gut microbiota structure at 92 and 132 gd differed compared to the other time points (Figure 3B; PERMANOVA, *p* < 0.05), reflecting the joint effect of strongyle infection, lower temperatures and increased pasture quality and abundance (Figure S3). Similarly, the correspondence analysis based on weighted Unifrac distance (Figure 3C) and the Jaccard network (Figure 3D) analyses suggested that the overall gut microbiota composition was largely similar between S and R ponies at each time point but differed from the 92 gd onward. In the same line, we did not detect significant differences between the two groups using Bray-Curtis similarity coefficients at the genus level. The stress values of the NMDS plots were high (0.207), and the cumulative inertia of the two first axes of the CA was low (13.22%) indicating that the changes were very small (Figure S9).

Even if the overall α- and β-diversity did not differ significantly between groups following the infection, changes in relative abundance of certain genera concomitantly arose with strongyle egg excretion at 92 gd. Genera of the unclassified *Ruminococcaceae* and *Eubacteriaceae* families as well as very low abundant genera (e.g., *Paludibacter, Campylobacter, Bacillus, Pseudomonas, Clostridium* III, *Acetivibrio)* increased in S relative to R ponies (adjusted *p* ≤ 0.25; Figure 4). Moreover, the relative abundance of *Acetivibrio*, and *Clostridium* III highly correlated with FEC (Pearson correlation coefficient ρ > 0.60). This genera enrichment occurred simultaneously with a reduction of dominant genera such as *Ruminococcus, Clostridium* XIVa but also members of the *Lachnospiraceae* family (adjusted *p* ≤ 0.25, Figure 4). However, none of the differences observed at 92 gd were sustained until 132 gd. The complete list of differentially expressed genera, *p*-values and adjusted *p*-values is presented in the Table S3.

The shifts in the gut bacterial composition between S and R at 92 gd were predicted to cause functional modifications, as inferred from PICRUSt (Table S4). Functional predictions showed an enrichment trend in S ponies microbiota (adjusted *p* < 0.10) for the mineral absorption, protein digestion and absorption, as well as of some of the pathways related to cell motility (e.g., bacterial chemotaxis, bacterial motility proteins, flagellar assembly), lipid metabolism (sphingolipid metabolism), peroxisome, and signal transduction (phosphatidylinositol signaling system) among others compared to the R group at 92 gd (Table S4).

The gut microbiota co-occurrence networks were marginally different between S and R ponies at 92 gd (Figure S10). The topological properties were calculated to describe the complex pattern of inter-relationships among nodes, and to distinguish differences in taxa correlations between these two groups of ponies (Table S5). The structural properties of the S network were slightly greater than the R network, indicating more connections and closer relationships of microbial taxa in the S group. Notably, S co-occurrence network displayed higher levels of betweenness centrality (which measures the number of shortest paths going through a given node) and higher degree levels (which describes the number of neighbors) relative to R network. Nodes with the highest degree and betweenness centrality values were identified as key genera in the co-occurrence networks. Key genera in S ponies were related to the Clostridiales order (e.g., *Clostridium* IV, *Roseburia, Nakamurella*) or Spirochaetales (e.g., *Treponema*), whereas key genera in the R network included members of Clostridiales order (e.g., *Clostridium* XIV, *Roseburia*) and Bacteroidales (e.g., *Alloprevotella*).

Interestingly, natural parasite infection increased the concentration of protozoa in S ponies at 92 gd (Figure 5B), but did not alter bacterial concentrations (Figure 5C) and fecal pH between the pony groups within each time point (Figure 5D). The concentration of anaerobic fungi was higher in the S group compared to R group at day 92 (Figure 5A).

## Discussion

While alternative strategies are needed for a more sustainable control of horse strongyle infection, the factors contributing to the over-dispersed distribution of these parasites in their hosts remain poorly characterized (Wood et al., 2012; Kornaś et al., 2015; Debeffe et al., 2016). In their preferred niche, strongyles are surrounded by gut microbiota, and reciprocal interactions between them are expected. Our study aimed to identify the consequences of parasite infection on the gut microbiota and host physiology under natural conditions and to seek a microbiological signature of strongyle infection in resistant and susceptible ponies.

For the first time, we have characterized how gut microbiological communities and host biochemical and hematological parameters varied between resistant and susceptible ponies under natural parasite infection conditions. Eosinophilia and high levels of monocytes were observed in resistant animals, while minor changes were found in the gut microbiological composition. Therefore, it seems that horses with FEC within the range of values reported herein, i.e., 0 to 800 eggs/g, do not exhibit major perturbations of their gut microbiota structure and composition. While the relationship between horse welfare and the severity of strongyle infection remains unclear, this finding may provide extra support for the current FEC cut-offs (200 or 500 eggs per gram) used to decide if treatment is needed or not (Nielsen et al., 2010).

Under our experimental setting, the first 43 days of the trial were heavily marked by both a heat wave that caused reduced pasture yield and quality, and a transition from an indoor to an outdoor management system. The combination of these factors induced a mixture of different physiological stresses for the ponies over these 43 days. Under these challenging climatic and nutritional conditions, R ponies displayed higher levels of eosinophils and monocytes in comparison to the S ponies. Eosinophilia has been reported in experimentally challenged horses (Murphy and Love, 1997). However, no larvae were recovered from pasture samples in our study suggesting mild, if any, contamination, in line with the drought conditions that are detrimental to their survival (Nielsen et al., 2007). In addition, the mild residual egg excretion observed after 24 grazing days occurred in only two susceptible ponies, which cannot explain the increased level of eosinophils in the R group. Therefore, this differential profile in immune cell populations may result from the stressful environmental conditions as previously reported (Collier et al., 2008).

The constituent phyla, families, and genera within the gut microbiota of R and S ponies during this first 43 days of the experiment were congruent with other studies performed on horses (Costa et al., 2012, 2015; Shepherd et al., 2012; Steelman et al., 2012; Dougal et al., 2013; Weese et al., 2015; Mach et al., 2017; Venable et al., 2017).

Differences in the microbiota structure and composition during the transition period were noted not between S and R individuals but rather between 4 and 6 years old individuals, suggesting that the gut microbiota of individuals at different ages who have varying effective immune response (Lyons et al., 1999) react to abiotic stressors differently. Extreme heat temperatures and consequent nutrient scarcity in the pasture might have been sufficient to cause a transient alteration in cortisol release patterns in horses (Aurich et al., 2015) and consequently shift the gut microbiota composition (reviewed by Clark and Mach, 2016). Differences in grazing behavior between individuals might also have played a role in shaping the gut microbiota. This has been observed in other animals such as sheep in which some individuals within a herd favor less N-rich swards of grass in order to reduce the rate of ingestion of parasitic larvae (Hutchings et al., 2000).

Notably, our results showed a difference in anaerobic fungal and protozoan concentrations between S and R ponies during the transition period. As these differences were found from the beginning of the experiment, we could not establish a relationship with any of the biotic or abiotic stressors occurring during this transition period. Nevertheless, these observations suggest that a potential intrinsic resistance to strongyle infection might be associated with reduced anaerobic fungal concentrations and increased protozoan concentrations. A larger scale survey linking together estimated breeding values for strongyle infection and microbiological structure could help resolve this hypothesis. In ruminants, Hsu et al. (1991) reported a reduction in ruminal fungal zoospores in individuals with higher protozoan concentrations possibly as a result of protozoal predation and competition for nutrients. Clearly, more studies are needed to fully understand the interactions between anaerobic fungi, protozoa and the susceptibility to parasite infection. However, indisputably, individuals with different susceptibility to parasite infection present different anaerobic fungal and protozoan concentrations under worm-free conditions.

**Figure 6:**
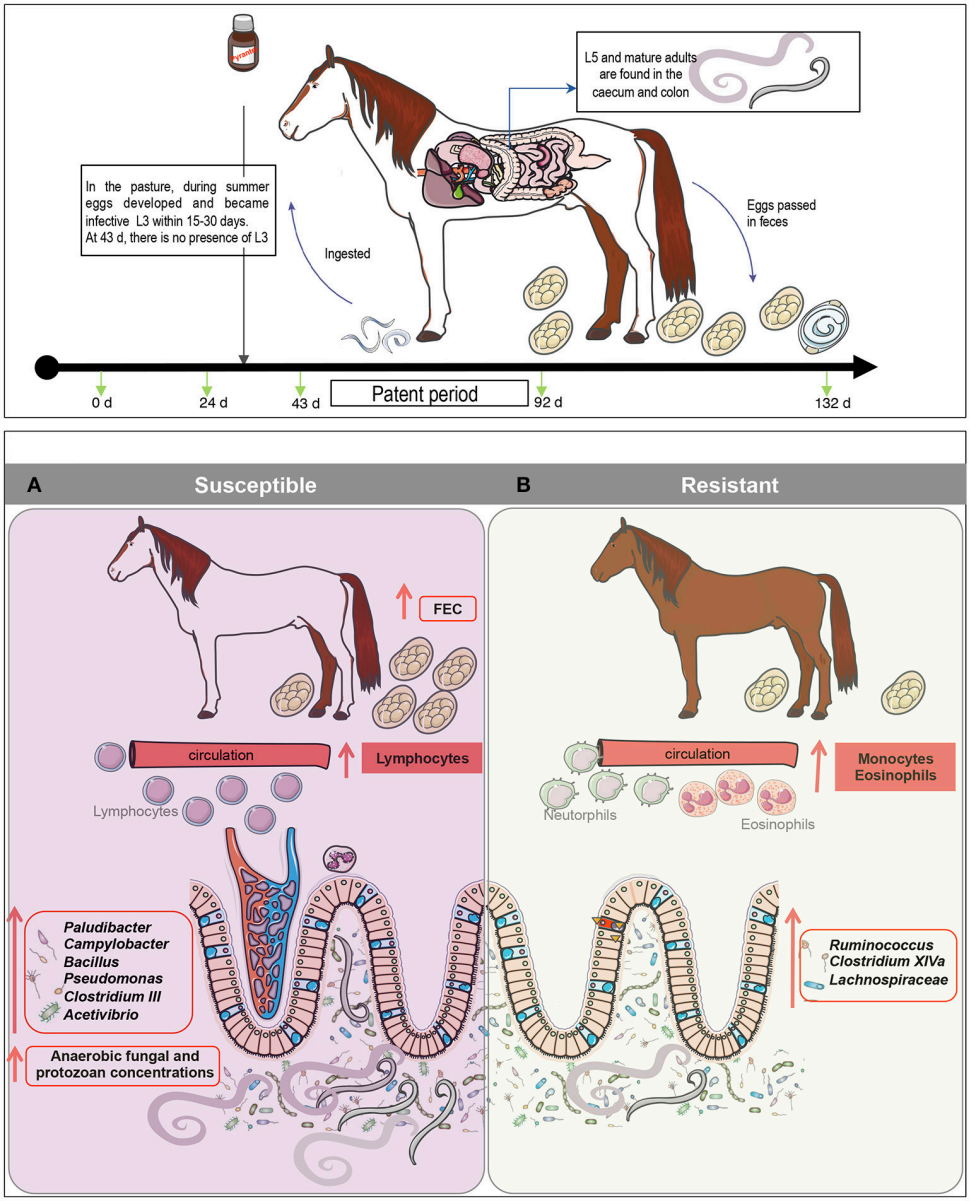
A model for gut microbiota modifications and their effects on host physiology after natural strongyle infection. We hypothesize that natural parasite infection in susceptible ponies increases lymphocyte levels but decreases monocyte and eosinophil cell counts. **(A)** Concomitantly, parasite infection induced alterations in bacterial-anaerobic fungal-protozoal inter-kingdom, increasing the abundance of *Paludibacter, Campylobacter, Bacillus, Pseudomonas, Clostridium* III, *Acetivibrio* and the overall concentrations of anaerobic fungi, protozoa and parasite egg counts in the feces. **(B)** On the other hand, butyrate producing bacteria such as members of *Ruminococcus, Clostridium* XIVa and *Lachnospiraceae* family were found to be depleted in susceptible ponies, but enriched in resistant animals, suggesting a possible effect of N-butyrate on the protection of inflammation in resistant animals. Because butyrate is a potent inhibitor of inflammation, it is suggested that susceptible ponies are prone to the gut inflammation because of the altered abundance of butyrate-producing bacteria.

The most interesting findings brought forward by this work were obtained during the natural strongyle infection from day 43 to 132 of the experiment (Figure 6A). Congruent with our initial hypothesis, the observed FEC matched the predicted resistance levels throughout the trial, hence supporting the high reproducibility of FEC (Debeffe et al., 2016; Scheuerle et al., 2016) and the feasibility to select for more resistant individuals (Kornaś et al., 2015). Eosinophil and monocyte counts remained higher in R compared to S ponies, particularly at 132 gd, whereas lymphocytes counts continued higher in S individuals. Eosinophils are the primary effectors of a Th_2_ cell response during parasite infection (Weller and Spencer, 2017). Previous studies in horses infected with cyathostomins showed a Th_2_ polarization during infection (Davidson et al., 2005) and an enrichment of eosinophils at the site of infection (Thamsborg et al., 1998; Collobert-Laugier et al., 2002). Interestingly, genetically resistant breeds of sheep also displayed higher levels of eosinophils at the site of infection (Terefe et al., 2009). However, more detailed immunological characterization under controlled conditions would be needed as our data are likely obscured by confounding variation in environmental factors (e.g., extreme heat temperatures and decrease of pasture quality before the parasite infection).

Despite the limited parasite infection level monitored throughout the experiment, FEC differences between S and R ponies at 92 gd were slightly reflected in the gut bacterial composition and predicted function. However, the estimated α- and β-diversity analysis suggested no differences in the overall microbiota structure between S and R individuals. These might be related to the fact that the resolution of taxonomical classification of sequences based on a limited segment of the 16S rRNA gene, such as the V3-V4 region, is relatively low (Jovel et al., 2016). One of the approaches to increase the resolution of taxonomical classification would be assemble individuals' reads into larger fragments, technically known as contigs, which are more amenable for taxonomic classifications (Jovel et al., 2016).

Strongyle natural infection in S ponies coincided with an increase in pathobionts, such as *Pseudomonas, Campylobacter*, and *Bacillus* and a decrease in commensal genera such as *Clostridium* XIVa, *Ruminococcus*, and members of the unclassified *Lachnospiraceae* family (Figure 6B). Firmicutes belonging to the families *Ruminococcaceae* (also referred as clostridial cluster IV) and *Lachnospiraceae* (also referred as clostridial cluster XIVa) comprise mostly of the butyrate-producing bacteria in the human gut (Geirnaert et al., 2017). Due to butyrate's anti-inflammatory properties, it could be suggested that the higher level of helminth infection in susceptible ponies altered the abundance of butyrate-producing bacteria, which therefore modulated inflammation in the gut (Li et al., 2016). Additionally, the reduced abundance of *Clostridium* XIV in S ponies could have had functional importance such as immune protection against the overgrowth of pathobionts like *Pseudomonas* and *Campylobacter*. Furthermore, it is notable that a significant number of predicted bacterial functional pathways in S ponies reflected immunological mechanisms, including pathogen sensing, changes in lipids, and activation of intracellular signal transduction pathways that are critical for immune system regulation and maintaining energy homeostasis (Vassart and Costagliola, 2011). Although the mechanisms of the interactions between these specific gut genera and the inferred functional attributes require further elucidation (e.g., shotgun metagenomics), our findings suggest that specific modulations of the gut microbiota might be an effective strategy for managing parasite infections in horse.

The shifts in the bacterial structure and composition at 92 gd due to the presence of parasites in the gut lumen were associated with changes in protozoan concentrations between groups. Protozoan concentrations increased in S ponies at 92 gd, which could have had an important physiological consequence, especially in host carbohydrate metabolism (Dougal et al., 2012), or have an unclear clinical significance. It could be that strongyle infection makes the large intestine a more suitable environment for commensal protozoa. It has been observed in chimpanzees that a positive correlation between intestinal parasites and commensal protozoa arose from complex interactions mediated by the host immune response and competition among taxa (McLennan et al., 2017). In horses, Güiris et al. (2010) have shown that helminths cohabit with commensal protozoa in the gastrointestinal tract in natural conditions. In line with the seemingly symbiotic relationship between *H. polygyrus* and bacteria from the family *Lactobacillaceae* in mice (Reynolds et al., 2015), it could be speculated that strongyle and protozoa may contribute to each other's success in the ecological niche of the equine intestines. Variations in protozoan concentrations might also reflect host adaptations for nutrient accessibility and acquisition during infection. In fact, protozoa have an important role in pectin degradation although have a limited role in cellulose digestion (Julliand et al., 1999). The key contribution of *Treponema*, a non-pathogenic carbohydrate metabolizing bacteria (Han et al., 2011), to the co-occurrence network of S ponies at 92 gd also suggests that compensatory mechanisms were induced to degrade fiber and supply the host with micro and macronutrients needed to face the infection. It is well-known that micronutrient deficiencies as well as numerous other non-dietary mechanisms, such as inflammation, disruption of the gut barrier integrity and even viral, fungal or parasite overgrowth might dictate the microbial-microbial as well as microbial-environmental interactions within the gut (Mach and Clark, 2017).

In conclusion, we have showed that predicted resistant ponies did excrete fewer eggs than their susceptible counterparts, further advocating for FEC measurement in the field to guide anthelminthic usage. This differential susceptibility status was also associated with divergent immunological responses and slight differences in microbiological composition under worm-free conditions that could be markers of resistance status but needs further validation.

Resistance was associated with high levels of circulating eosinophils and monocytes and lower levels of lymphocytes; however, the modifications of gut microbiota composition were modest. During parasite infection, susceptible ponies presented a decrease in known butyrate producing bacteria, such as *Ruminococcus, Clostridium* XIVa and members of the *Lachnospiraceae* family, which may have led to a disruption of mucosal homeostasis, intestinal inflammation and dysbiosis. In line with this hypothesis, an increase in pathobionts such as *Pseudomonas* and *Campylobacter* were observed in susceptible ponies as well as changes in several predicted immunological pathways. Moreover, these bacterial modifications in S ponies at day 92 occurred simultaneously with an increase in protozoan concentrations. Strongyles and these microbial taxa may hence contribute to each other's success or S ponies might favor the increase of these carbohydrate-degrading microorganisms to enhance the supply of micro and macronutrients needed to face the strongyle infection.

Our results therefore suggest that susceptibility to strongyle infection occurs in the presence of moderate gut microbiological factors that affect individual risk. This investigation should be followed up by experimental work in order to establish the causative reasons for variations in the gut microbiome observed in this study, as well as SCFA levels assessed due to their role in gut health.

## Author contributions

GS, AB, and NM designed the experiment. AC, GS, and NM drafted the main manuscript text. NM designed and carried out the bioinformatics and biostatistical analyses, prepared all the figures and provided critical feedback on content. VB performed the RT-qPCR analyses. FR was in charge of pony maintenance and care throughout the experiment and managed sampling. AM analyzed the chemical composition of the diet. JC and CK performed fecal egg counts. MR performed the blood analysis. All authors reviewed the manuscript and approved the final version.

### Conflict of interest statement

The author AB is currently employed by the company Pancosma SA (CH-1218 Geneva, Switzerland). The other authors declare that the research was conducted in the absence of any commercial or financial relationships that could be construed as a potential conflict of interest.

## Supplementary material

Figure S1 Genetic kinship between the 20 Welsh ponies in the experiment. **(A)** Dot plot of the historical egg counts (eggs/g feces) in each of the susceptible (S) and resistant (R) animals included in the study since 2011; **(B)** Density plot of historical fecal egg counts values in S and R animals. In all cases, S individuals are indicated in purple color and R individuals in green. **(C)** Pedigree plot. A six-generation pedigree plot is illustrated, with different shapes for male (squares) and female (circles). The shapes are black for the 20 ponies in the study; **(D)** Heatmap of the kinship coefficient matrix, which assess the genetic resemblance between ponies. Each entry in the matrix is the kinship coefficient between two subjects. Animals are arranged in the order of their genetic relatedness. Genetically similar animals are near each other. Note that the diagonal elements did not have values above unity, showing no consanguineous mating in the families. The treatment (S or R) is delineated below to the animal name. In the heatmap, red = high values of genetic relatedness, white = low values of genetic relatedness.

Figure S2 Overview of the data analysis in the study. Effect of parasite susceptibility on gut microbiota composition, gut-related parameters and host variables across time. Step 1: Measurement of the gut microbiota composition between susceptible (S) and resistant (R) ponies across time. This step involved the analysis of the α-diversity and β-diversity between S and R ponies across time, as well as the analysis to assess gut bacterial genera whose relative abundances changed between groups across time, the prediction of the corresponding KEGG pathways, and the inference of the co-occurrence network. Step 2: Measurement of the fecal parasite eggs counts and the gut related parameters between S and R ponies across time. The gut parameters included the anaerobic fungal, bacterial and protozoan concentrations, as well as the pH in the feces. Step 3: Measurement of the host variables between S and R ponies across time. The host parameters included the body weight, the daily average gain, and the hematological and biochemical parameters in blood.

Figure S3 Weather and pasture quality throughout the experiment. **(A)** Daily precipitation and temperatures recorded at a meteorological station located 14 km from the experimental pasture. Maximum and minimum daily temperatures are colored in red and blue, respectively. Dashed line represents precipitation; **(B)** Chemical composition (crude protein (CP), neutral and acid detergent fiber (NDF and ADF), crude fiber (CF), crude ash, and acid detergent lignin (ADL) of hay at day 0 and pasture at day 24, 43, 92, and 132. From day 1 to day 43, the lack of rainfall resulted in significant soil moisture deficits and reduced growth rates of pasture. This period coincided with the late-flowering stage of the pasture, when stems and leaves are being depleted of nutrients and herbage maturation and lignification increases. After day 43, the environmental conditions were eminently favorable to start a second pasture cycle, with a quick herbage growth, high protein content and lower fiber content. We observed that herbage protein peaked at day 92, after the intense fall rains and the increased senescence of green material.

Figure S4 Hemogram data in susceptible and resistant animals across time. The evaluation of the hemogram involved the determination of the hematocrit, total white blood cell counts, total erythrocyte count, erythrocyte indices and platelet counts. The determination of the total white blood cells **(A)** was performed between S (purple boxes) and R (green boxes) animals across time. The quantification of different type of leukocytes: neutrophils **(B)** and basophils **(C)** were described between S and R animals across time. The values corresponding to red blood cell distribution width **(D)**, microcytic **(E)** and macrocytic **(F)** platelets, as well as microcytic red blood cells **(G)**, and macrocytic red blood cells **(H)** were plotted. In all cases, boxes show median and interquartile range, and whiskers indicate 5th to 95th percentile. ^*^*p* < 0.05 for comparison between S and R ponies in each time point.

Figure S5 Top 20 most abundant genera in susceptible and resistant animals across time. Area plot representation of the 20 most abundant genera in feces of susceptible (S) and resistant (R) animals across time. In each plot, the non-colored space represents the total proportion of the minor genera grouped together.

Figure S6 Animal performance between susceptible and resistant animals across time. **(A)** Boxplot and violin plot representation of body weight (kg) between susceptible (S) and resistant (R) ponies across time; **(B)** Boxplot and violin plot representation of average daily gain (kg) between S and R ponies across time. In all cases, S animals were colored in purple and R animals in green. ^*^*p* < 0.05 for comparison between S and R ponies in each time point.

Figure S7 Biochemical data in susceptible and resistant animals across time. Levels of albumin **(A)**, cholesterol **(B)**, globins **(C)**, glucose **(D)**, alkaline phosphatase **(E)**, ratio albumin/goblins **(F)**, total proteins **(G)** and urea **(H)** in susceptible (S, purple boxes) and resistant animals (R green boxes) were plotted. Boxes show median and interquartile range, and whiskers indicate 5th to 95th percentile. ^*^*p* < 0.05 for comparison between S and R ponies in each time point.

Figure S8 Estimation of α-diversity indexes (observed species richness, Chao1 and Shannon) from the OTU table between time points. Estimation of the α-diversity indexes across the experiment. The box color indicates the time point analyzed: (dark blue = 0 days, cyan = 24 days, pink = 43 days, green = 92 days, and yellow = 132 days). A Dunn test was performed as *post-hoc* analysis to determine which levels of the independent variable differ from each other level. For all variables with the same letter, the difference between the means is not statistically significant (*p* > 0.05). If two variables have different letters, they are significantly different (*p* < 0.05).

Figure S9 Estimation of β-diversity indexes from genera table in susceptible and resistant animals across the experiment. **(A)** Non-metric multidimensional scaling (NMDS) of Bray Curtis distances to compare fecal communities at the level of genera that differ between susceptible (S) and resistant (R) animals across the experiment. Microbial communities were not significantly different among R and S treatments represented by convex hulls. Distance between objects on the plot represents relative dissimilarity. Stress >0.2 indicates that differences should be interpreted with caution; **(B)** Correspondence analyses of Bray-Curtis distances to compare fecal communities at the level of genera that differ between S and R animals across the experiment. Both CA axes 1 and 2 were plotted. Together they explained 13.22% of whole inertia. The convex hulls color indicates the treatment group: S = purple and R = green.

Figure S10 Co-occurrence genera network at 92 days after the entry to the pasture for susceptible and resistant ponies. The correlations among genera were calculated using the PCIT method, which identifies significant co-occurrence patterns. The size of the node is proportional to genera abundance. Node fill color corresponds to phylum taxonomic classification. Edges color represent positive (red) and negative (blue) connections, the edge thickness is equivalent to the correlation values. Only genera with a relative abundance >0.10 were included.

Table S1 Summary of study samples and fecal bacterial 16S rRNA gene amplicon sequence datasets.

Table S2 The OTU taxonomical assignments and OTU counts in each pony across time.

Table S3 Differences in relative abundance of genera between susceptible (S) and resistant (R) animals across time.

Table S4 Differences in predicted KEGG pathway abundance between susceptible (S) and resistant (R) animals across time. Predicted pathways values across time are represented as %, where normalized counts from a particular pathway is divides by the total number of counts in each sample.

Table S5 Topological properties of co-occurring networks obtained from susceptible and resistant animals at day 92 of the experiment.

## Abbreviations

ADF: acid detergent fiber
ADL: acid detergent lignin
AFNOR: Association Française de Normalization
ANOSIM: analysis of similarities
ANOVA: Analysis of the variance
CA: correspondence analysis
DM: dry matter
FEC: fecal egg counts
KEGG: Kyoto Encyclopedia of Genes and Genomes
NDCS: neutral detergent soluble carbohydrate
NDF: neutral detergent fiber
NFC: Non-fiber carbohydrate
NMDS: non-parametric multidimensional scaling
OTU: operational taxonomic units
PCIT: partial correlation and information theory
PCoA: principal coordinates analysis
PERMANOVA: Permutational Multivariate Analysis of Variance
PICRUSt: phylogenetic investigation of communities by reconstruction of unobserved states
QIIME: Quantitative Insights Into Microbial Ecology
qPCR: real time quantitative PCR
RDP: Ribosomal Database Project naïve Bayesian classifier
SCFA: short chain fatty acids.

## References

[B1] AFNOR (1977a). Dosage de L'azote en vue du Calcul de la Teneur en Protéines Brutes. Norme Française NF V18-100. Paris: Afnor.

[B2] AFNOR (1977b). Dosage des Cendres Brutes. Norme Française NF V18-101. Paris: Afnor.

[B3] AFNOR (1982). Détermination de la Teneur en eau. Norme Française NF V18-109. Paris: Afnor.

[B4] AFNOR (1993). Produits Agricoles et Alimentaires. Détermination de la Cellulose Brute-Méthode Générale. Norme Française NF V03-040. Paris: Afnor.

[B5] AFNOR (1997). Détermination Séquentielle des Constituants Pariétaux. Norme Française NF V18-122. Paris: Afnor.

[B6] Aurich J. Wulf M. Ille N. Erber R. von Lewinski M. Palme R.. (2015). Effects of season, age, sex, and housing on salivary cortisol concentrations in horses. Domest. Anim. Endocrinol. 52, 11–16. 10.1016/j.domaniend.2015.01.003 25700267

[B7] Boyett D. Hsieh M. H. (2014). Wormholes in host defense: how helminths manipulate host tissues to survive and reproduce. PLoS Pathog. 10:e1004014. 10.1371/journal.ppat.1004014 24743351 PMC3990715

[B8] Bucknell D. Gasser R. Beveridge I. (1995). The prevalence and epidemiology of gastrointestinal parasites of horses in Victoria, Australia. Int. J. Parasitol. 25, 711–724. 10.1016/0020-7519(94)00214-9 7657457

[B9] Caporaso J. G. Kuczynski J. Stombaugh J. Bittinger K. Bushman F. D. Costello E. K. (2010). QIIME allows analysis of high- throughput community sequencing data Intensity normalization improves color calling in SOLiD sequencing. Nat. Publ. Gr. 7, 335–336. 10.1038/nmeth.f.303 PMC3156573 20383131

[B10] Cattadori I. M. Sebastian A. Hao H. Katani R. Albert I. Eilertson K. E.. (2016). Impact of helminth infections and nutritional constraints on the small intestine microbiota. PLoS ONE 11:e0159770. 10.1371/journal.pone.0159770 27438701 PMC4954658

[B11] Chao A. S. J. (1984). Nonparametric estimation of the number of classes in a population. Scand. J. Stat. 11, 256–270.

[B12] Clark A. Mach N. (2016). Exercise-induced stress behavior, gut-microbiota-brain axis and diet: a systematic review for athletes. J. Int. Soc. Sports Nutr. 13:43. 10.1186/s12970-016-0155-6 27924137 PMC5121944

[B13] Cole J. R. Wang Q. Cardenas E. Fish J. Chai B. Farris R. J.. (2009). The ribosomal database project: improved alignments and new tools for rRNA analysis. Nucleic Acids Res. 37, 141–145. 10.1093/nar/gkn879 19004872 PMC2686447

[B14] Collier R. J. Collier J. L. Rhoads R. P. Baumgard L. H. (2008). Invited review: genes involved in the bovine heat stress response. J. Dairy Sci. 91, 445–454. 10.3168/jds.2007-0540 18218730

[B15] Collobert-Laugier C. Hoste H. Sevin C. Chartier C. Dorchies P. (2002). Mast cell and eosinophil mucosal responses in the large intestine of horses naturally infected with cyathostomes. Vet. Parasitol. 107, 251–264. 10.1016/S0304-4017(02)00119-X 12127254

[B16] Corning S. (2009). Equine cyathostomins: a review of biology, clinical significance and therapy. Parasit. Vectors 2(Suppl. 2):S1. 10.1186/1756-3305-2-S2-S1 19778462 PMC2751837

[B17] Costa M. C. Arroyo L. G. Allen-Vercoe E. Stämpfli H. R. Kim P. T. Sturgeon A.. (2012). Comparison of the fecal microbiota of healthy horses and horses with colitis by high throughput sequencing of the V3-V5 region of the 16s rRNA gene. PLoS ONE 7:e41484. 10.1371/journal.pone.0041484 22859989 PMC3409227

[B18] Costa M. C. Silva G. Ramos R. V. Staempfli H. R. Arroyo L. G. Kim P.. (2015). Characterization and comparison of the bacterial microbiota in different gastrointestinal tract compartments in horses. Vet. J. 205, 74–80. 10.1016/j.tvjl.2015.03.018 25975855

[B19] D'Elia R. DeSchoolmeester M. L. Zeef L. A. H. Wright S. H. Pemberton A. D. Else K. J. (2009). Expulsion of *Trichuris muris* is associated with increased expression of angiogenin 4 in the gut and increased acidity of mucins within the goblet cell. BMC Genomics 10:492. 10.1186/1471-2164-10-492 19852835 PMC2774869

[B20] Davidson A. J. Hodgkinson J. E. Proudman C. J. Matthews J. B. (2005). Cytokine responses to Cyathostominae larvae in the equine large intestinal wall. Res. Vet. Sci. 78, 169–176. 10.1016/j.rvsc.2004.07.005 15563925

[B21] Debeffe L. McLoughilin P. D. Medill S. A. Stewart K. Andres D. Shurty T.. (2016). Negative covariance between parasite load and body condition in a population of feral horses. Parasitology 143, 983–997. 10.1017/S0031182016000408 27046508

[B22] DeSantis T. Z. Hugenholtz P. Larsen N. Rojas M. Brodie E. L. Keller K.. (2006). Greengenes, a chimera-checked 16S rRNA gene database and workbench compatible with ARB. Appl. Environ. Microbiol. 72, 5069–5072. 10.1128/AEM.03006-05 16820507 PMC1489311

[B23] Dixon P. (2003). VEGAN, a package of R functions for community ecology. J. Veg. Sci. 14, 927 10.1111/j.1654-1103.2003.tb02228.x

[B24] Dougal K. de la Fuente G. Harris P. A. Girdwood S. E. Pinloche E. Newbold C. J. (2013). Identification of a core bacterial community within the large intestine of the horse. PLoS ONE 8:e77660. 10.1371/journal.pone.0077660 24204908 PMC3812009

[B25] Dougal K. Harris P. A. Edwards A. Pachebat J. A. Blackmore T. M. Worgan H. J.. (2012). A comparison of the microbiome and the metabolome of different regions of the equine hindgut. FEMS Microbiol. Ecol. 82, 642–652. 10.1111/j.1574-6941.2012.01441.x 22757649

[B26] Duarte A. M. Jenkins T. P. Latrofa M. S. Giannelli A. Papadopoulos E. De Carvalho L. M. (2016). Helminth infections and gut microbiota-A feline perspective. Parasit. Vectors 9, 1–9. 10.1186/s13071-016-1908-4 27912797 PMC5135779

[B27] Edgar R. C. (2010). Search and clustering orders of magnitude faster than BLAST. Bioinformatics 26, 2460–2461. 10.1093/bioinformatics/btq461 20709691

[B28] Edgar R. C. Haas B. J. Clemente J. C. Quince C. Knight R. (2011). UCHIME improves sensitivity and speed of chimera detection. Bioinformatics 27, 2194–2200. 10.1093/bioinformatics/btr381 21700674 PMC3150044

[B29] El-Ashram S. Suo X. (2017). Exploring the microbial community (microflora) associated with ovine Haemonchus contortus (macroflora) field strains. Sci. Rep. 7:70 10.1038/s41598-017-00171-2. 28250429 PMC5427911

[B30] Ericsson A. C. Johnson P. J. Lopes M. A. Perry S. C. Lanter H. R. (2016). A microbiological map of the healthy equine gastrointestinal tract. PLoS ONE 11:e0166523. 10.1371/journal.pone.0166523 27846295 PMC5112786

[B31] Fernandes J. Su W. Rahat-Rozenbloom S. Wolever T. M. S. Comelli E. M. (2014). Adiposity, gut microbiota and faecal short chain fatty acids are linked in adult humans. Nutr. Diabetes 4:e121. 10.1038/nutd.2014.23 24979150 PMC4079931

[B32] Fricke W. F. Song Y. Wang A.-J. Smith A. Grinchuk V. Pei C. (2015). Type 2 immunity-dependent reduction of segmented filamentous bacteria in mice infected with the helminthic parasite Nippostrongylus brasiliensis. Microbiome 3:40 10.1186/s40168-015-0103-8 26377648 PMC4574229

[B33] Geirnaert A. Calatayud M. Grootaert C. Laukens D. Devriese S. Smagghe G.. (2017). Butyrate-producing bacteria supplemented *in vitro* to Crohn's disease patient microbiota increased butyrate production and enhanced intestinal epithelial barrier integrity. Sci. Rep. 7:11450. 10.1038/s41598-017-11734-8 28904372 PMC5597586

[B34] Geurden T. van Doorn D. Claerebout E. Kooyman F. De Keersmaecker S. Vercruysse J.. (2014). Decreased strongyle egg re-appearance period after treatment with ivermectin and moxidectin in horses in Belgium, Italy and The Netherlands. Vet. Parasitol. 204, 291–296. 10.1016/j.vetpar.2014.04.013 24880643

[B35] Giacomin P. Agha Z. Loukas A. (2016). Helminths and intestinal flora team up to improve gut health. Trends Parasitol. 32, 664–666. 10.1016/j.pt.2016.05.006 27234811

[B36] Giles C. J. Urquhart K. A. Longstaffe J. A. (1985). Larval cyathostomiasis (immature trichonema induced enteropathy): a report of 15 clinical cases. Equine Vet. J. 17, 196–201. 10.1111/j.2042-3306.1985.tb02469.x 4076127

[B37] Goodrich J. K. Waters J. L. Poole A. C. Sutter J. L. Koren O. Blekhman R.. (2014). Human genetics shape the gut microbiome. Cell 159, 789–799. 10.1016/j.cell.2014.09.053 25417156 PMC4255478

[B38] Gruner L. Raynaud J. P. (1980). Technique allégée de prélèvements d'herbe et de numération, pour juger de l'infestation des pâturages de bovins par les larves de nématodes parasites. Med. Vet. 131, 521–529.

[B39] Güiris A. D. M. Rojas H. N. M. Berovides A. V. Sosa P. J. Pérez E. M. E. Cruz A. E. (2010). Biodiversity and distribution of helminths and protozoa in naturally infected horses from the biosphere reserve “La Sierra Madre de Chiapas”, México. Vet. Parasitol. 170, 268–277. 10.1016/j.vetpar.2010.02.016. 20307938

[B40] Han C. Gronow S. Teshima H. Lapidus A. Nolan M. Lucas S. (2011). Complete genome sequence of Treponema succinifaciens type strain (6091T). Stand. Genomic Sci. 4, 361–370. 10.4056/sigs.1984594 21886863 PMC3156407

[B41] Holm J. B. Sorobetea D. Kiilerich P. Ramayo-Caldas Y. Estellé J. Ma T.. (2015). Chronic Trichuris muris infection decreases diversity of the intestinal microbiota and concomitantly increases the abundance of lactobacilli. PLoS ONE 10:e0125495. 10.1371/journal.pone.0125495 25942314 PMC4420551

[B42] Houlden A. Hayes K. S. Bancroft A. J. Worthington J. J. Wang P. Grencis R. K.. (2015). Chronic Trichuris muris infection in C57BL/6 mice causes significant changes in host microbiota and metabolome: effects reversed by pathogen clearance. PLoS ONE 10:e0125945. 10.1371/journal.pone.0125945 25938477 PMC4418675

[B43] Hsu J. Fahey G. Merchen N. Mackie R. (1991). Effects of defaunation and various nitrogen supplementation regimens on microbial numbers and activity in the rumen of sheep. J. Anim. Sci. 69, 1279–1289. 10.2527/1991.6931279x 2061256

[B44] Hutchings M. Kyriazakis I. Papachristou T. Gordon I. Jackson F. (2000). The herbivores' dilemma: trade-offs between nutrition and parasitism in foraging decisions. Oecologia 124, 242–251. 10.1007/s004420000367. 28308185

[B45] Jovel J. Patterson J. Wang W. Hotte N. O'Keefe S. Mitchel T. (2016). Characterization of the gut microbiome using 16S or shotgun metagenomics. Front. Microbiol. 7:459 10.3389/fmicb.2016.00459 27148170 PMC4837688

[B46] Julliand V. De Vaux A. Millet L. Fonty G. (1999). Identification of Ruminococcus flavefaciens as the predominant cellulolytic bacterial species of the equine caecum. Appl. Environ. Microbiol. 65, 3738–3741. 10427077 10.1128/aem.65.8.3738-3741.1999 PMC91562

[B47] Kornaś S. Sallé G. Skalska M. David I. Ricard A. Cabaret J. (2015). Estimation of genetic parameters for resistance to gastro-intestinal nematodes in pure blood Arabian horses. Int. J. Parasitol. 45, 237–242. 10.1016/j.ijpara.2014.11.003 25592965

[B48] Kuzmina T. A. Dzeverin I. Kharchenko V. A. (2016). Strongylids in domestic horses: influence of horse age, breed and deworming programs on the strongyle parasite community. Vet. Parasitol. 227, 56–63. 10.1016/j.vetpar.2016.07.024 27523938

[B49] Langille M. G. I. Zaneveld J. Caporaso J. G. McDonald D. Knights D. Reyes J. A.. (2013). Predictive functional profiling of microbial communities using 16S rRNA marker gene sequences. Nat. Biotechnol. 31, 814–821. 10.1038/nbt.2676 23975157 PMC3819121

[B50] Lee S. C. Tang M. S. Lim Y. A. L. Choy S. H. Kurtz Z. D. Cox L. M.. (2014). Helminth colonization is associated with increased diversity of the gut microbiota. PLoS Negl. Trop. Dis. 8:e2880. 10.1371/journal.pntd.0002880 24851867 PMC4031128

[B51] Lester H. E. Bartley D. J. Morgan E. R. Hodgkinson J. E. Stratford C. H. Matthews J. B. (2013). A cost comparison of faecal egg count-directed anthelmintic delivery versus interval programme treatments in horses. Vet. Rec. 173:371. 10.1136/vr.101804 24068698

[B52] Li R. W. Li W. Sun J. Yu P. Baldwin R. L. Urban J. F. (2016). The effect of helminth infection on the microbial composition and structure of the caprine abomasal microbiome. Sci. Rep. 6:20606. 10.1038/srep20606 26853110 PMC4757478

[B53] Li R. Wu S. Li W. Navarro K. Couch R. Hill D.. (2012). Alterations in the porcine colon microbiota induced by the gastrointestinal nematode *Trichuris suis*. Infect. Immun. 80, 2150–2157. 10.1128/IAI.00141-12 22493085 PMC3370577

[B54] Li R. W. Wu S. Li W. Huang Y. Gasbarre L. C. (2011). Metagenome plasticity of the bovine abomasal microbiota in immune animals in response to ostertagia ostertagi infection. PLoS ONE 6:e24417. 10.1371/journal.pone.0024417 21931709 PMC3170331

[B55] Lichtenfels J. R. Kharchenko V. A. Dvojnos G. M. (2008). Illustrated identification keys to strongylid parasites (*Strongylidae: Nematoda*) of horses, zebras and asses (Equidae). Vet. Parasitol. 156, 4–161. 10.1016/j.vetpar.2008.04.026 18603375

[B56] Lim M. Y. You H. J. Yoon H. S. Kwon B. Lee J. Y. Lee S.. (2017). The effect of heritability and host genetics on the gut microbiota and metabolic syndrome. Gut 66, 1031–1038. 10.1136/gutjnl-2015-311326 27053630

[B57] Lluch J. Servant F. Païssé S. Valle C. Valière S. Kuchly C.. (2015). The characterization of novel tissue microbiota using an optimized 16S metagenomic sequencing pipeline. PLoS ONE 10:e0142334. 10.1371/journal.pone.0142334 26544955 PMC4636327

[B58] Love S. Murphy D. Mellor D. (1999). Pathogenicity of cyathostome infection. Vet. Parasitol. 85, 113–121. 10.1016/S0304-4017(99)00092-8 10485358

[B59] Lozupone C. Stomabaugh J. Gordon J. Jansson J. Knight R. (2012). Diversity, stability and resilience of the human gut microbiota. Nature 489, 220–230. 10.1038/nature11550 22972295 PMC3577372

[B60] Lyons E. T. Tolliver S. C. Drudge J. H. (1999). Historical perspective of cyathostomes: prevalence, treatment and control programs. Vet. Parasitol. 85, 97–112. 10.1016/S0304-4017(99)00091-6 10485357

[B61] Mach N. Clark A. (2017). Micronutrient deficiencies and the human gut microbiota. Trends Microbiol. 25, 607–610. 10.1016/j.tim.2017.06.004 28645724

[B62] Mach N. Foury A. Kittelmann S. Reigner F. Moroldo M. Ballester M.. (2017). The effects of weaning methods on gut microbiota composition and horse physiology. Front. Physiol. 8:535. 10.3389/fphys.2017.00535 28790932 PMC5524898

[B63] Mackie R. I. Wilkins C. A. (1988). Enumeration of anaerobic bacterial microflora of the equine gastrointestinal tract. Appl. Environ. Microbiol. 54, 2155–2160. 3190223 10.1128/aem.54.9.2155-2160.1988 PMC202828

[B64] Marley M. G. Meganathan R. Bentley R. (1986). Menaquinone (vitamin K2) biosynthesis in *Escherichia coli*: synthesis of o-succinylbenzoate does not require the decarboxylase activity of the ketoglutarate dehydrogenase complex. Biochemistry 25, 1304–1307. 10.1021/bi00354a017 3516220

[B65] Matthews J. B. (2014). Anthelmintic resistance in equine nematodes. Int. J. Parasitol. Drugs Drug Resist. 4, 310–315. 10.1016/j.ijpddr.2014.10.003 25516842 PMC4266799

[B66] McKenney E. A. Williamson L. Yoder A. D. Rawls J. F. Bilbo S. D. Parker W. (2015). Alteration of the rat cecal microbiome during colonization with the helminth *Hymenolepis diminuta*. Gut Microbes 6, 182–193. 10.1080/19490976.2015.1047128 25942385 PMC4615828

[B67] McLennan M. R. Hasegawa H. Bardi M. Huffman M. A. (2017). Gastrointestinal parasite infections and selfmedication in wild chimpanzees surviving in degraded forest fragments within an agricultural landscape mosaic in Uganda. PLoS ONE 12:e0180431. 10.1371/journal.pone.0180431 28692673 PMC5503243

[B68] Mcmurdie P. J. Holmes S. (2012). Analysis of high-throughput phylogenetic sequence data. Pac. Symp. Biocomput. 235–246. 22174279 PMC3357092

[B69] Midha A. Schlosser J. Hartmann S. (2017). Reciprocal interactions between nematodes and their microbial environments. Front. Cell. Infect. Microbiol. 7:144. 10.3389/fcimb.2017.00144 28497029 PMC5406411

[B70] Murphy D. Love S. (1997). The pathogenic effects of experimental cyathostome infections in ponies. Vet. Parasitol. 70, 99–110. 10.1016/S0304-4017(96)01153-3 9195714

[B71] Navas-Molina J. Peralta-Sánchez J. M. González A. McMurdie P. J. Vázquez-Baeza Y. (2013). Advancing our understanding of the human microbiome using QIIME. Methods Enzymol. 74, 371–444. 10.1016/B978-0-12-407863-5.00019-8. PMC4517945 24060131

[B72] Nedjadi T. Moran A. W. Al-Rammahi M. a Shirazi-Beechey, S. P. (2014). Characterization of butyrate transport across the luminal membranes of equine large intestine. Exp. Physiol. 99, 1335–1347. 10.1113/expphysiol.2014.077982 25172888

[B73] Nicholson J. K. Holmes E. Kinross J. Burcelin R. Gibson G. Jia W. (2012). Host-gut microbiota metabolic interactions. Science 108, 1262–1268. 10.1126/science.1223813 22674330

[B74] Nielsen M. K. Fritzen B. Duncan J. L. Guillot J. Eysker M. Dorchies P.. (2010). Practical aspects of equine parasite control: a review based upon a workshop discussion consensus. Equine Vet. J. 42, 460–468. 10.1111/j.2042-3306.2010.00065.x 20636785

[B75] Nielsen M. K. Kaplan R. M. Thamsborg S. M. Monrad J. Olsen S. N. (2007). Climatic influences on development and survival of free-living stages of equine strongyles: implications for worm control strategies and managing anthelmintic resistance. Vet. J. 174, 23–32. 10.1016/j.tvjl.2006.05.009 16815051

[B76] Nielsen M. K. Vidyashankar A. N. Olsen S. N. Monrad J. Thamsborg S. M. (2012). Strongylus vulgaris associated with usage of selective therapy on Danish horse farms-is it reemerging? Vet. Parasitol. 189, 260–266. 10.1016/j.vetpar.2012.04.039 22703964

[B77] Ogbourne C. (1976). The prevalence, relative abundance and site distribution of nematodes of the subfamily Cyathostominae in horses killed in Britain. J. Helminthol. 50, 203–214. 10.1017/S0022149X00027760 993579

[B78] Peachey L. E. Jenkins T. P. Cantacessi C. (2017). This gut ain't big enough for both of us. or is it? Helminth-microbiota interactions in veterinary species. Trends Parasitol. 33, 619–632. 10.1016/j.pt.2017.04.004 28506779

[B79] Price M. N. Dehal P. S. Arkin A. P. (2010). FastTree 2-approximately maximum-likelihood trees for large alignments. PLoS ONE 5:e9490. 10.1371/journal.pone.0009490 20224823 PMC2835736

[B80] Ramayo-Caldas Y. Mach N. Lepage P. Levenez F. Denis C. Lemonnier G.. (2016). Phylogenetic network analysis applied to pig gut microbiota identifies an ecosystem structure linked with growth traits. ISME J. 10, 2973–2977. 10.1038/ismej.2016.77 27177190 PMC5148198

[B81] Raynaud J. P. (1970). Etude de l'efficacité d'une technique de coproscopie quantitative pour le diagnostic de routine et le controle des infestations parasitaires des bovins, ovins, equines et porcins. Ann. Parasitol. 45, 321–342. 5531507

[B82] Reinemeyer C. R. Prado J. C. Nielsen M. K. (2015). Comparison of the larvicidal efficacies of moxidectin or a five-day regimen of fenbendazole in horses harboring cyathostomin populations resistant to the adulticidal dosage of fenbendazole. Vet. Parasitol. 214, 100–107. 10.1016/j.vetpar.2015.10.003 26477278

[B83] Relf V. E. Morgan E. R. Hodgkinson J. E. Matthews J. B. (2013). Helminth egg excretion with regard to age, gender and management practices on UK Thoroughbred studs. Parasitology 140, 641–652. 10.1017/S0031182012001941 23351718

[B84] Reverter A. Chan E. K. F. (2008). Combining partial correlation and an information theory approach to the reversed engineering of gene co-expression networks. Bioinformatics 24, 2491–2497. 10.1093/bioinformatics/btn482 18784117

[B85] Reynolds L. A. Finlay B. B. Maizels R. M. (2015). Cohabitation in the intestine: interactions among Helminth parasites, bacterial microbiota, and host immunity. J. Immunol. 195, 4059–4066. 10.4049/jimmunol.1501432 26477048 PMC4617609

[B86] Reynolds L. A. Smith K. A. Filbey K. J. Harcus Y. Hewitson J. P. Redpath S. A.. (2014). Commensal-pathogen interactions in the intestinal tract lactobacilli promote infection with, and are promoted by, helminth parasites. Gut Microbes 5, 522–532. 10.4161/gmic.32155 25144609 PMC4822684

[B87] Rideout J. R. He Y. Navas-Molina J. A. Walters W. A. Ursell L. K. Gibbons S. M.. (2014). Subsampled open-reference clustering creates consistent, comprehensive OTU definitions and scales to billions of sequences. PeerJ 2:e545. 10.7717/peerj.545 25177538 PMC4145071

[B88] Sallé G. Cortet J. Bois I. Dubès C. Larrieu C. Landrin V. (2017). Risk factor analysis of equine strongyle resistance to anthelmintics. Int. J. Parasitol. Drugs Drug Resist. 7, 407–415. 10.1016/j.ijpddr.2017.10.007 29149701 PMC5727347

[B89] Sallé G. Cortet J. Koch C. Reigner F. Cabaret J. (2015). Economic assessment of FEC-based targeted selective drenching in horses. Vet. Parasitol. 214, 159–166. 10.1016/j.vetpar.2015.09.006 26414907

[B90] Scheuerle M. C. Stear M. J. Honeder A. Becher A. M. Pfister K. (2016). Repeatability of strongyle egg counts in naturally infected horses. Vet. Parasitol. 228, 103–107. 10.1016/j.vetpar.2016.08.021 27692309

[B91] Scott I. Bishop R. M. Pomroy W. E. (2015). Anthelmintic resistance in equine helminth parasites–a growing issue for horse owners and veterinarians in New Zealand? N. Z. Vet. J. 63, 188–198. 10.1080/00480169.2014.987840 25608588

[B92] Shannon C. E. (1997). The mathematical theory of communication. MD Comput 14, 306–317. 9230594

[B93] Shepherd M. L. Swecker W. S. Jensen R. V. Ponder M. A. (2012). Characterization of the fecal bacteria communities of forage-fed horses by pyrosequencing of 16S rRNA V4 gene amplicons. FEMS Microbiol. Lett. 326, 62–68. 10.1111/j.1574-6968.2011.02434.x 22092776

[B94] Smith M. A. Nolan T. J. Rieger R. Aceto H. Levine D. G. Nolen-Walston R.. (2015). Efficacy of major anthelmintics for reduction of fecal shedding of strongyle-type eggs in horses in the Mid-Atlantic region of the United States. Vet. Parasitol. 214, 139–143. 10.1016/j.vetpar.2015.09.025 26518644

[B95] Steelman S. M. Chowdhary B. P. Dowd S. Suchodolski J. Janečka J. E. (2012). Pyrosequencing of 16S rRNA genes in fecal samples reveals high diversity of hindgut microflora in horses and potential links to chronic laminitis. BMC Vet. Res. 8:231. 10.1186/1746-6148-8-231 23186268 PMC3538718

[B96] Su C. Su L. Li Y. Long S. R. Chang J. Zhang W.. (2017). Helminth-induced alterations of the gut microbiota exacerbate bacterial colitis. Mucosal. Immunol. 11, 144–157. 10.1038/mi.2017.20. 28352104 PMC5620113

[B97] Sugahara H. Odamaki T. Hashikura N. Abe F. Xiao J. (2015). Differences in folate production by bifidobacteria of different origins. Biosci. Microbiota Food Heal. 34, 87–93. 10.12938/bmfh.2015-003 26594608 PMC4654071

[B98] Taylor M. A. Coop R. L. Wall R. L. (2007). Veterinary Parasitology. Blackwell Publishing Available online at: https://www.wiley.com/en-us/Veterinary+Parasitology%2C+3rd+Edition-p-9781118687116

[B99] Terefe G. Lacroux C. Prévot F. Grisez C. Bergeaud J. P. Bleuart C.. (2009). Eosinophils in Haemonchus contortus-infected resistant and susceptible breeds of sheep: abomasal tissue recruitment and *in vitro* functional state. Vet. Parasitol. 165, 161–164. 10.1016/j.vetpar.2009.06.041 19733438

[B100] Thamsborg S. M. Leifsson P. S. Grøndahl C. Larsen M. Nansen P. (1998). Impact of mixed strongyle infections in foals after one month on pasture. Equine Vet. J. 30, 240–245. 10.1111/j.2042-3306.1998.tb04494.x 9622325

[B101] Van Soest P. J. Robertson J. B. Lewis B. A. (1991). Methods for dietary fiber, neutral detergent fiber, and nonstarch polysaccharides in relation to animal nutrition. J. Dairy Sci. 74, 3583–3597. 10.3168/jds.S0022-0302(91)78551-2 1660498

[B102] Vassart G. Costagliola S. (2011). G protein-coupled receptors: mutations and endocrine diseases. Nat. Rev. Endocrinol. 7, 362–372. 10.1038/nrendo.2011.20 21301490

[B103] Venable E. B. Fenton K. A. Braner V. M. Reddington C. E. Halpin M. J. Heitz S. A. (2017). Effects of feeding management on the equine cecal microbiota. J. Equine Vet. Sci. 49, 113–121. 10.1016/j.jevs.2016.09.010

[B104] Weese J. S. Holcombe S. J. Embertson R. M. Kurtz K. A. Roessner H. A. Jalali M.. (2015). Changes in the faecal microbiota of mares precede the development of post partum colic. Equine Vet. J. 47, 641–649. 10.1111/evj.12361 25257320

[B105] Weller P. F. Spencer L. A. (2017). Functions of tissue-resident eosinophils. Nat. Rev. Immunol. 17, 746–760. 10.1038/nri.2017.95 28891557 PMC5783317

[B106] Wood E. L. D. Matthews J. B. Stephenson S. Slote M. Nussey D. H. (2012). Variation in fecal egg counts in horses managed for conservation purposes: individual egg shedding consistency, age effects and seasonal variation. Parasitology 140, 115–128. 10.1017/S003118201200128X. 22894917

[B107] Wu S. Li R. W. Li W. Beshah E. Dawson H. D. Urban J. F. (2012). Worm Burden-dependent disruption of the porcine colon microbiota by trichuris suis infection. PLoS ONE 7:e35470. 10.1371/journal.pone.0035470 22532855 PMC3332011

[B108] Xiao L. Herd R. P. Majewski G. A. (1994). Comparative efficacy of moxidectin and ivermectin against hypobiotic and encysted cyathostomes and other equine parasites. Vet. Parasitol. 53, 83–90. 10.1016/0304-4017(94)90020-5 8091622

[B109] Zaiss M. M. Harris N. L. (2016). Interactions between the intestinal microbiome and helminth parasites. Parasite Immunol. 38, 5–11. 10.1111/pim.12274 26345715 PMC5019230

