# Supplementary figures and images for: Strongyle Infection and Gut Microbiota: Profiling of Resistant and Susceptible Horses Over a Grazing Season
Source: Front Physiol. 2018 Mar 21;9:272. doi: 10.3389/fphys.2018.00272 (PMC5871743; doi:10.3389/fphys.2018.00272)

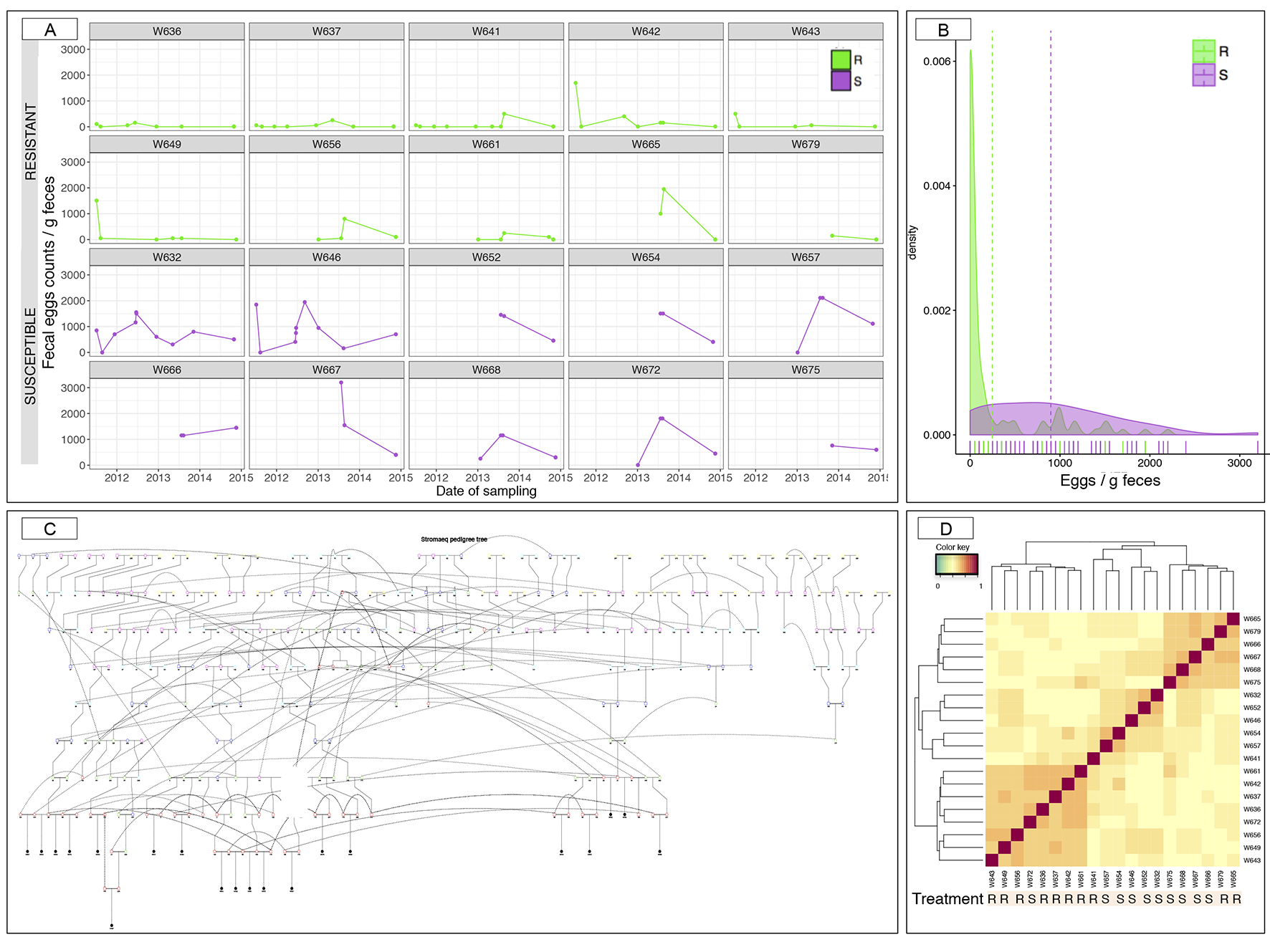

Supplement: Figure S1 — Genetic kinship between the 20 Welsh ponies in the experiment. (A) Dot plot of the historical egg counts (eggs/g feces) in each of the susceptible (S) and resistant (R) animals included in the study since 2011; (B) Density plot of historical fecal egg counts values in S and R animals. In all cases, S individuals are indicated in purple color and R individuals in green. (C) Pedigree plot. A six-generation pedigree plot is illustrated, with different shapes for male (squares) and female (circles). The shapes are black for the 20 ponies in the study; (D) Heatmap of the kinship coefficient matrix, which assess the genetic resemblance between ponies. Each entry in the matrix is the kinship coefficient between two subjects. Animals are arranged in the order of their genetic relatedness. Genetically similar animals are near each other. Note that the diagonal elements did not have values above unity, showing no consanguineous mating in the families. The treatment (S or R) is delineated below to the animal name. In the heatmap, red = high values of genetic relatedness, white = low values of genetic relatedness. [file Image1.TIF]

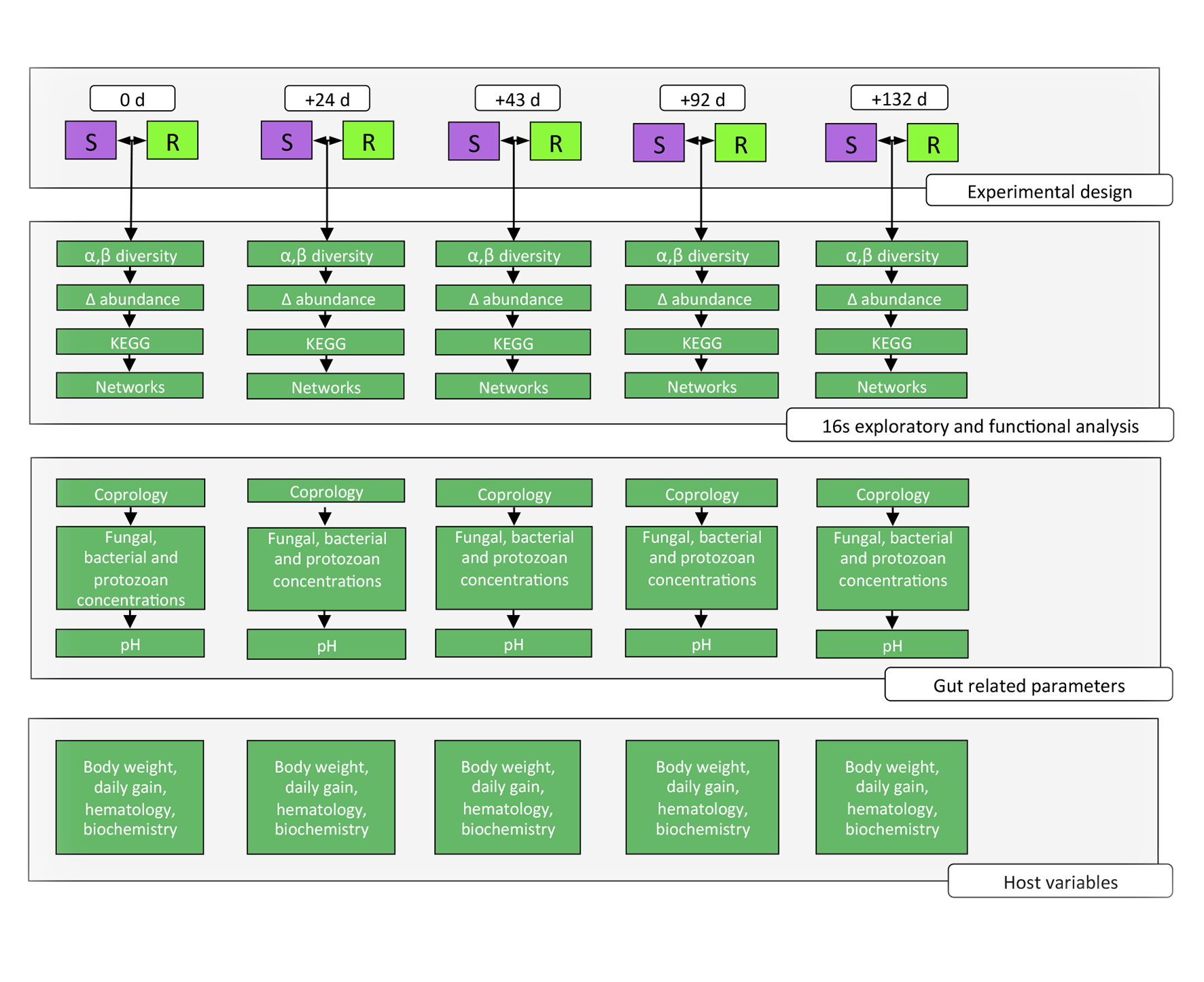

Supplement: Figure S2 — Overview of the data analysis in the study. Effect of parasite susceptibility on gut microbiota composition, gut-related parameters and host variables across time. Step 1: Measurement of the gut microbiota composition between susceptible (S) and resistant (R) ponies across time. This step involved the analysis of the α-diversity and β-diversity between S and R ponies across time, as well as the analysis to assess gut bacterial genera whose relative abundances changed between groups across time, the prediction of the corresponding KEGG pathways, and the inference of the co-occurrence network. Step 2: Measurement of the fecal parasite eggs counts and the gut related parameters between S and R ponies across time. The gut parameters included the anaerobic fungal, bacterial and protozoan concentrations, as well as the pH in the feces. Step 3: Measurement of the host variables between S and R ponies across time. The host parameters included the body weight, the daily average gain, and the hematological and biochemical parameters in blood. [file Image2.TIF]

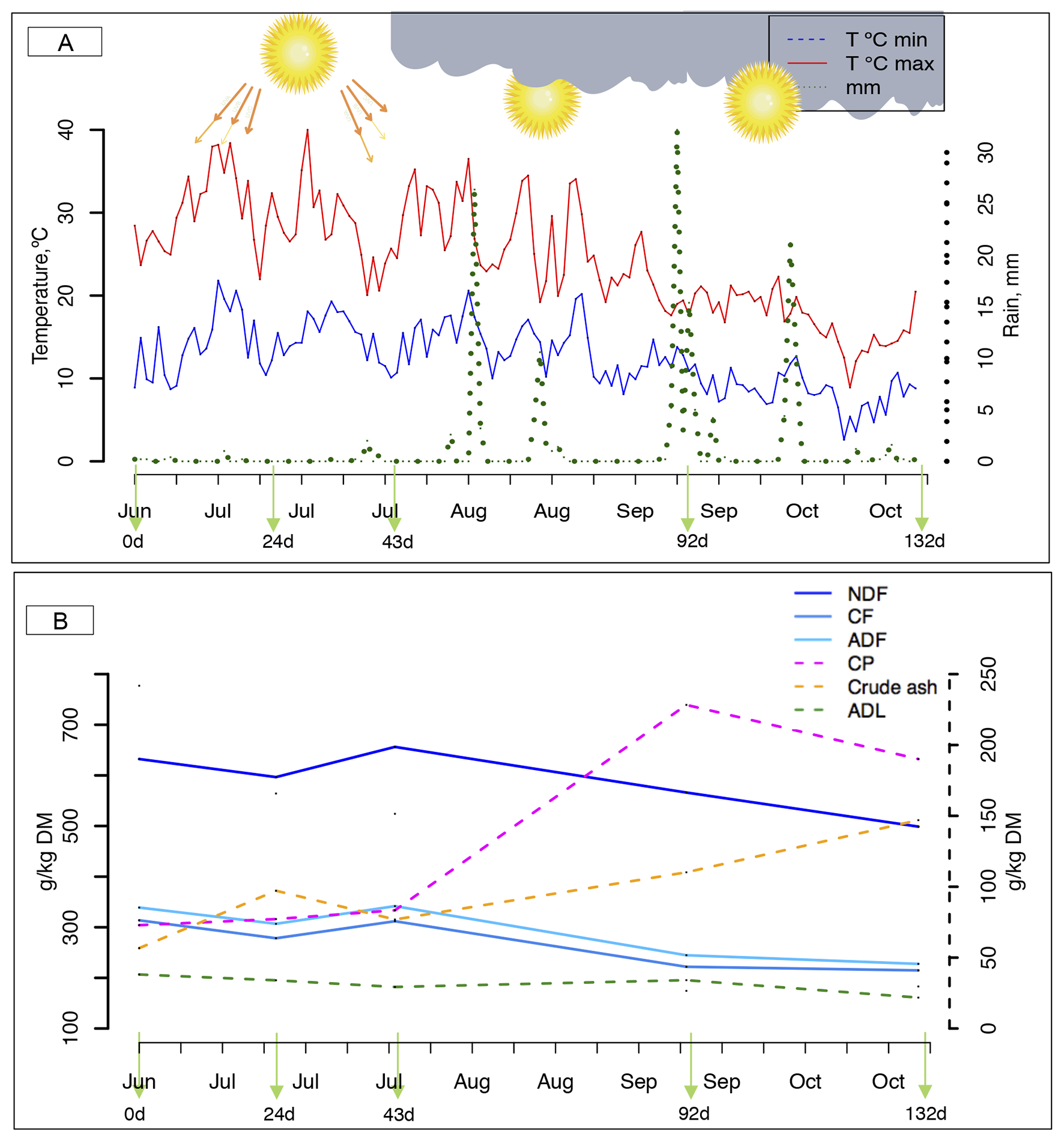

Supplement: Figure S3 — Weather and pasture quality throughout the experiment. (A) Daily precipitation and temperatures recorded at a meteorological station located 14 km from the experimental pasture. Maximum and minimum daily temperatures are colored in red and blue, respectively. Dashed line represents precipitation; (B) Chemical composition (crude protein (CP), neutral and acid detergent fiber (NDF and ADF), crude fiber (CF), crude ash, and acid detergent lignin (ADL) of hay at day 0 and pasture at day 24, 43, 92, and 132. From day 1 to day 43, the lack of rainfall resulted in significant soil moisture deficits and reduced growth rates of pasture. This period coincided with the late-flowering stage of the pasture, when stems and leaves are being depleted of nutrients and herbage maturation and lignification increases. After day 43, the environmental conditions were eminently favorable to start a second pasture cycle, with a quick herbage growth, high protein content and lower fiber content. We observed that herbage protein peaked at day 92, after the intense fall rains and the increased senescence of green material. [file Image3.TIF]

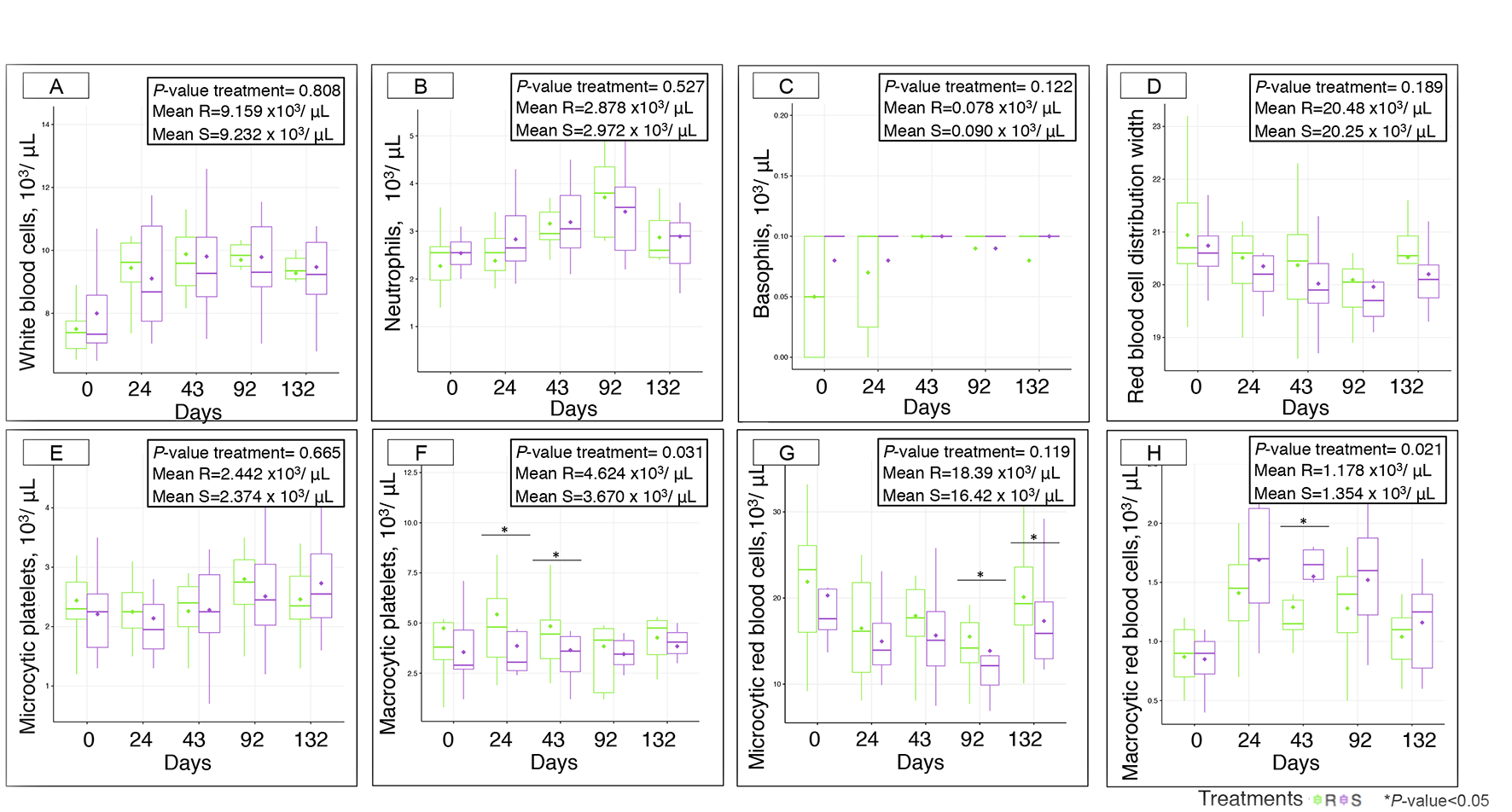

Supplement: Figure S4 — Hemogram data in susceptible and resistant animals across time. The evaluation of the hemogram involved the determination of the hematocrit, total white blood cell counts, total erythrocyte count, erythrocyte indices and platelet counts. The determination of the total white blood cells (A) was performed between S (purple boxes) and R (green boxes) animals across time. The quantification of different type of leukocytes: neutrophils (B) and basophils (C) were described between S and R animals across time. The values corresponding to red blood cell distribution width (D), microcytic (E) and macrocytic (F) platelets, as well as microcytic red blood cells (G), and macrocytic red blood cells (H) were plotted. In all cases, boxes show median and interquartile range, and whiskers indicate 5th to 95th percentile. *p < 0.05 for comparison between S and R ponies in each time point. [file Image4.TIF]

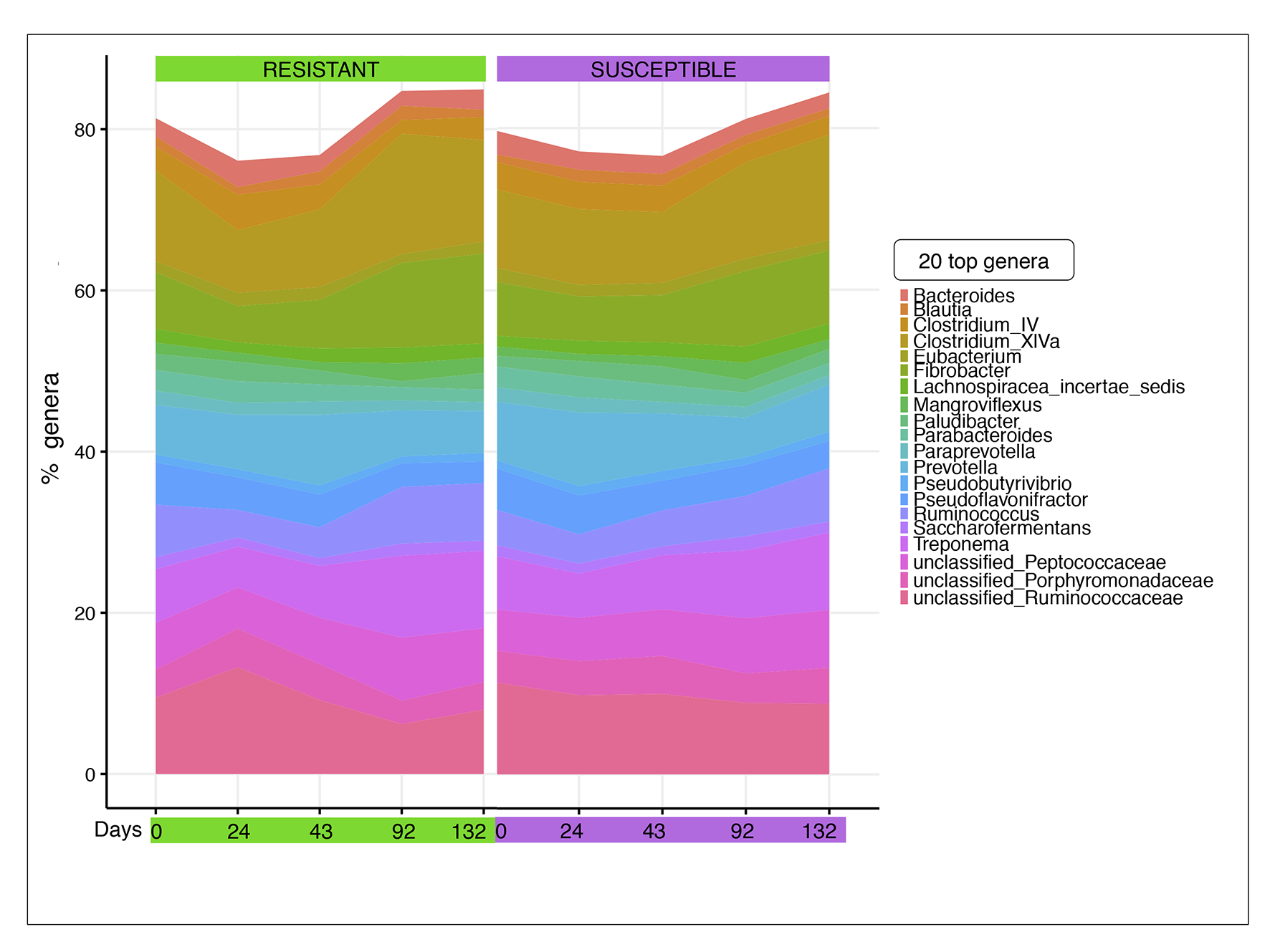

Supplement: Figure S5 — Top 20 most abundant genera in susceptible and resistant animals across time. Area plot representation of the 20 most abundant genera in feces of susceptible (S) and resistant (R) animals across time. In each plot, the non-colored space represents the total proportion of the minor genera grouped together. [file Image5.TIF]

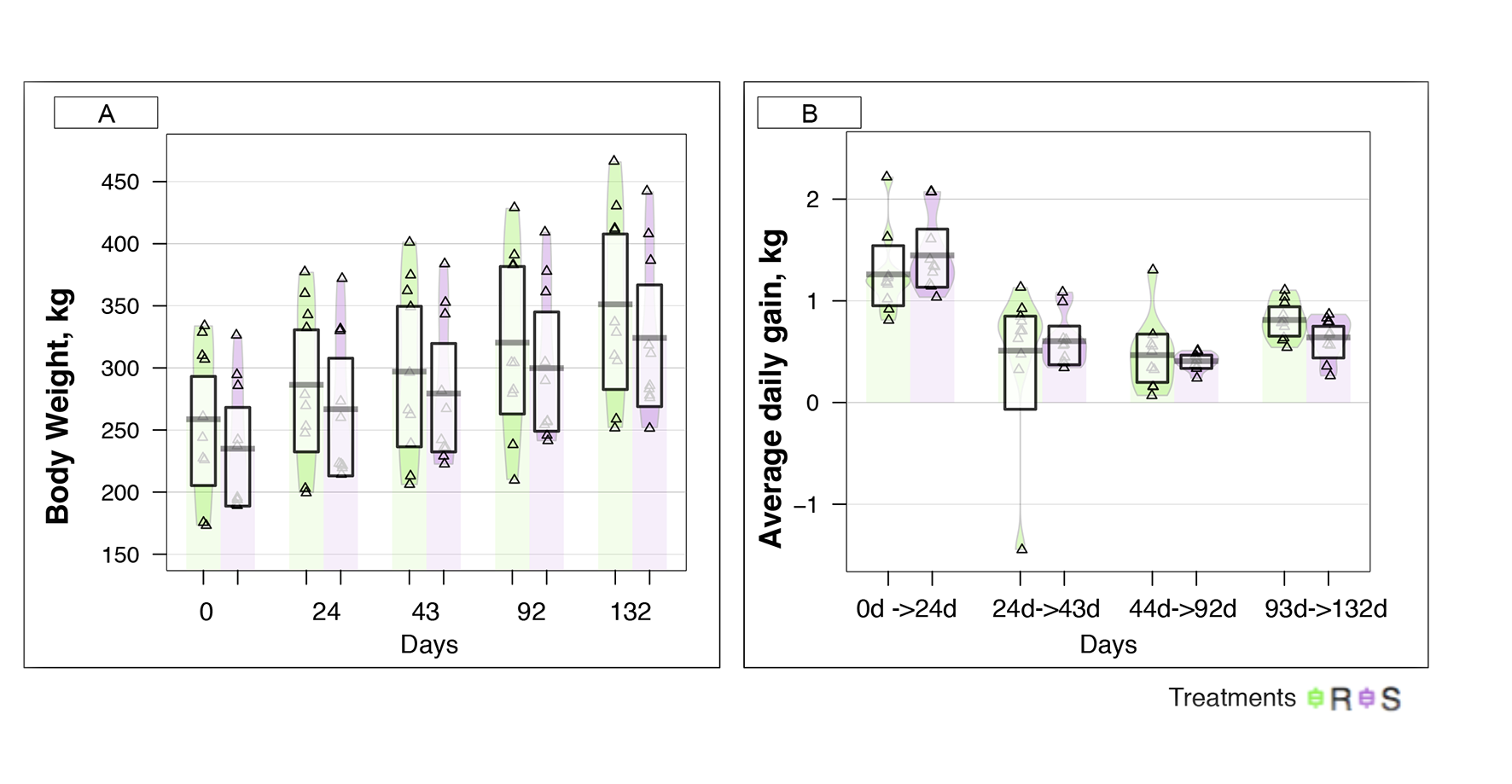

Supplement: Figure S6 — Animal performance between susceptible and resistant animals across time. (A) Boxplot and violin plot representation of body weight (kg) between susceptible (S) and resistant (R) ponies across time; (B) Boxplot and violin plot representation of average daily gain (kg) between S and R ponies across time. In all cases, S animals were colored in purple and R animals in green. *p < 0.05 for comparison between S and R ponies in each time point. [file Image6.TIF]

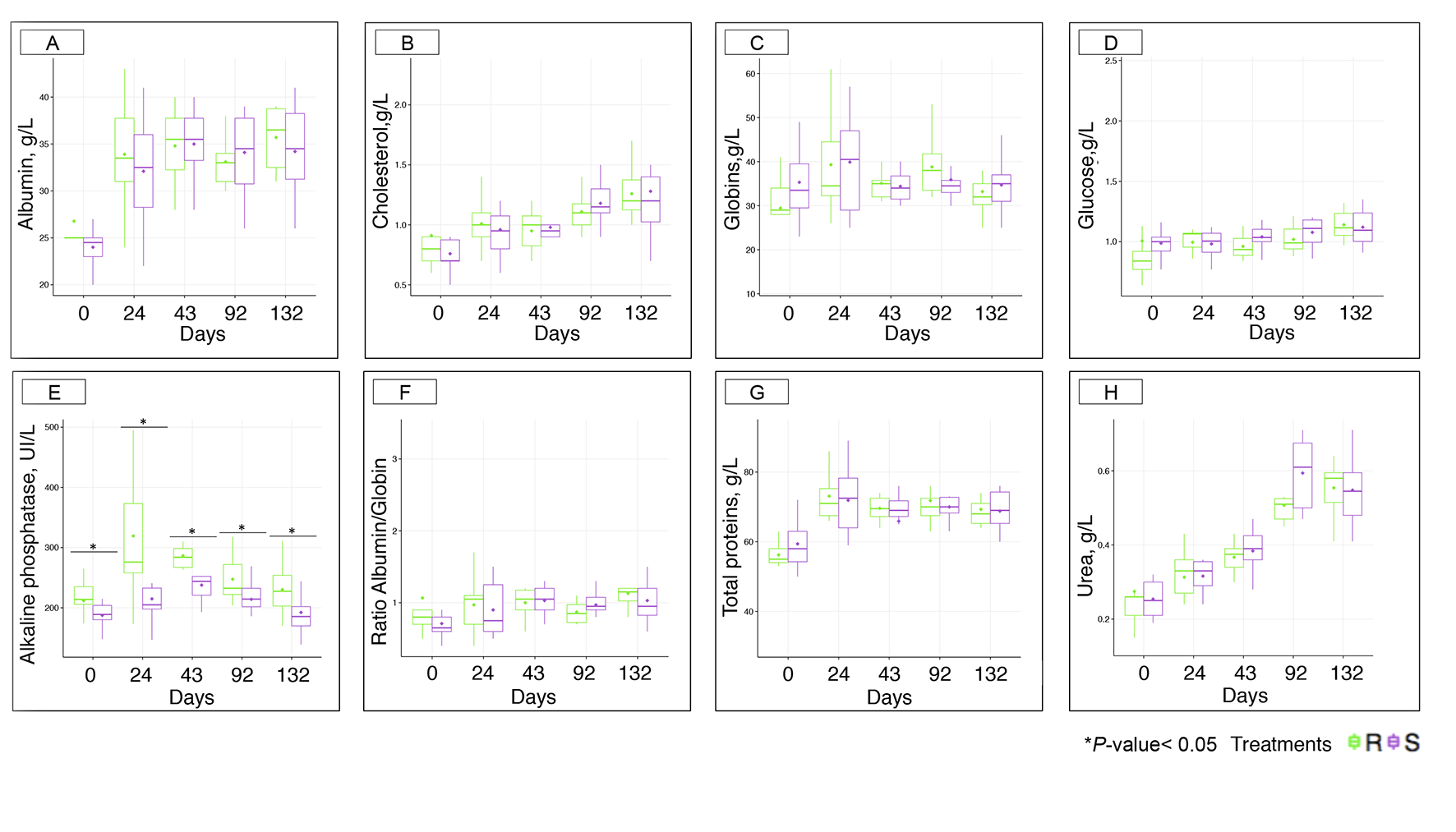

Supplement: Figure S7 — Biochemical data in susceptible and resistant animals across time. Levels of albumin (A), cholesterol (B), globins (C), glucose (D), alkaline phosphatase (E), ratio albumin/goblins (F), total proteins (G) and urea (H) in susceptible (S, purple boxes) and resistant animals (R green boxes) were plotted. Boxes show median and interquartile range, and whiskers indicate 5th to 95th percentile. *p < 0.05 for comparison between S and R ponies in each time point. [file Image7.TIF]

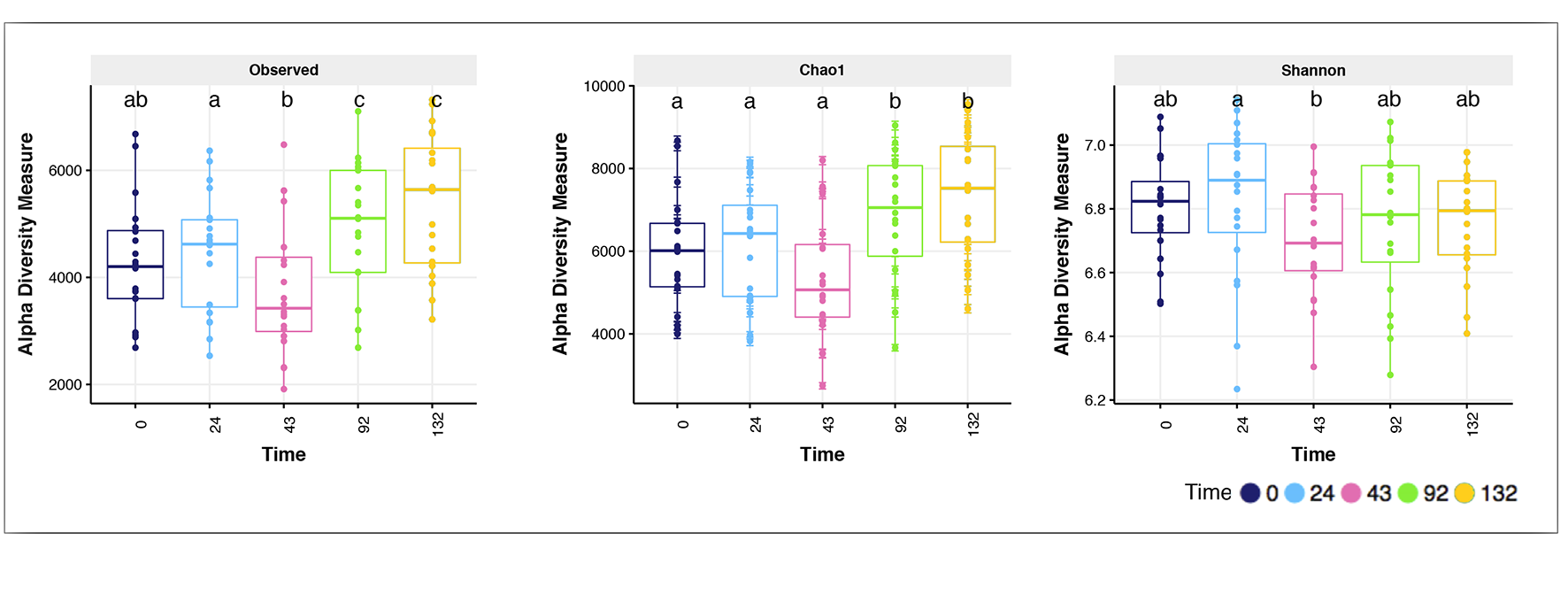

Supplement: Figure S8 — Estimation of α-diversity indexes (observed species richness, Chao1 and Shannon) from the OTU table between time points. Estimation of the α-diversity indexes across the experiment. The box color indicates the time point analyzed: (dark blue = 0 days, cyan = 24 days, pink = 43 days, green = 92 days, and yellow = 132 days). A Dunn test was performed as post-hoc analysis to determine which levels of the independent variable differ from each other level. For all variables with the same letter, the difference between the means is not statistically significant (p > 0.05). If two variables have different letters, they are significantly different (p < 0.05). [file Image8.TIF]

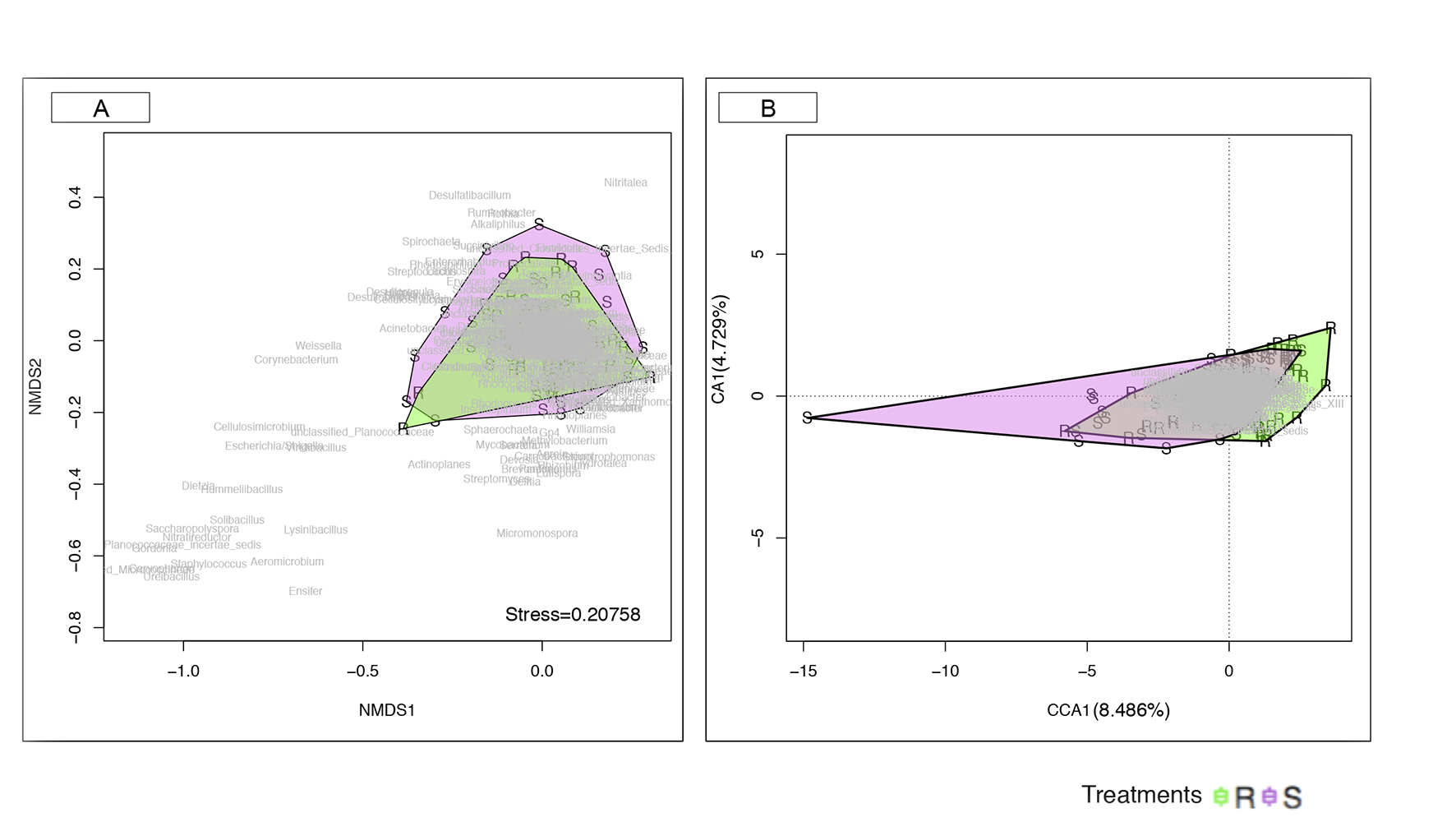

Supplement: Figure S9 — Estimation of β-diversity indexes from genera table in susceptible and resistant animals across the experiment. (A) Non-metric multidimensional scaling (NMDS) of Bray Curtis distances to compare fecal communities at the level of genera that differ between susceptible (S) and resistant (R) animals across the experiment. Microbial communities were not significantly different among R and S treatments represented by convex hulls. Distance between objects on the plot represents relative dissimilarity. Stress >0.2 indicates that differences should be interpreted with caution; (B) Correspondence analyses of Bray-Curtis distances to compare fecal communities at the level of genera that differ between S and R animals across the experiment. Both CA axes 1 and 2 were plotted. Together they explained 13.22% of whole inertia. The convex hulls color indicates the treatment group: S = purple and R = green. [file Image9.TIF]

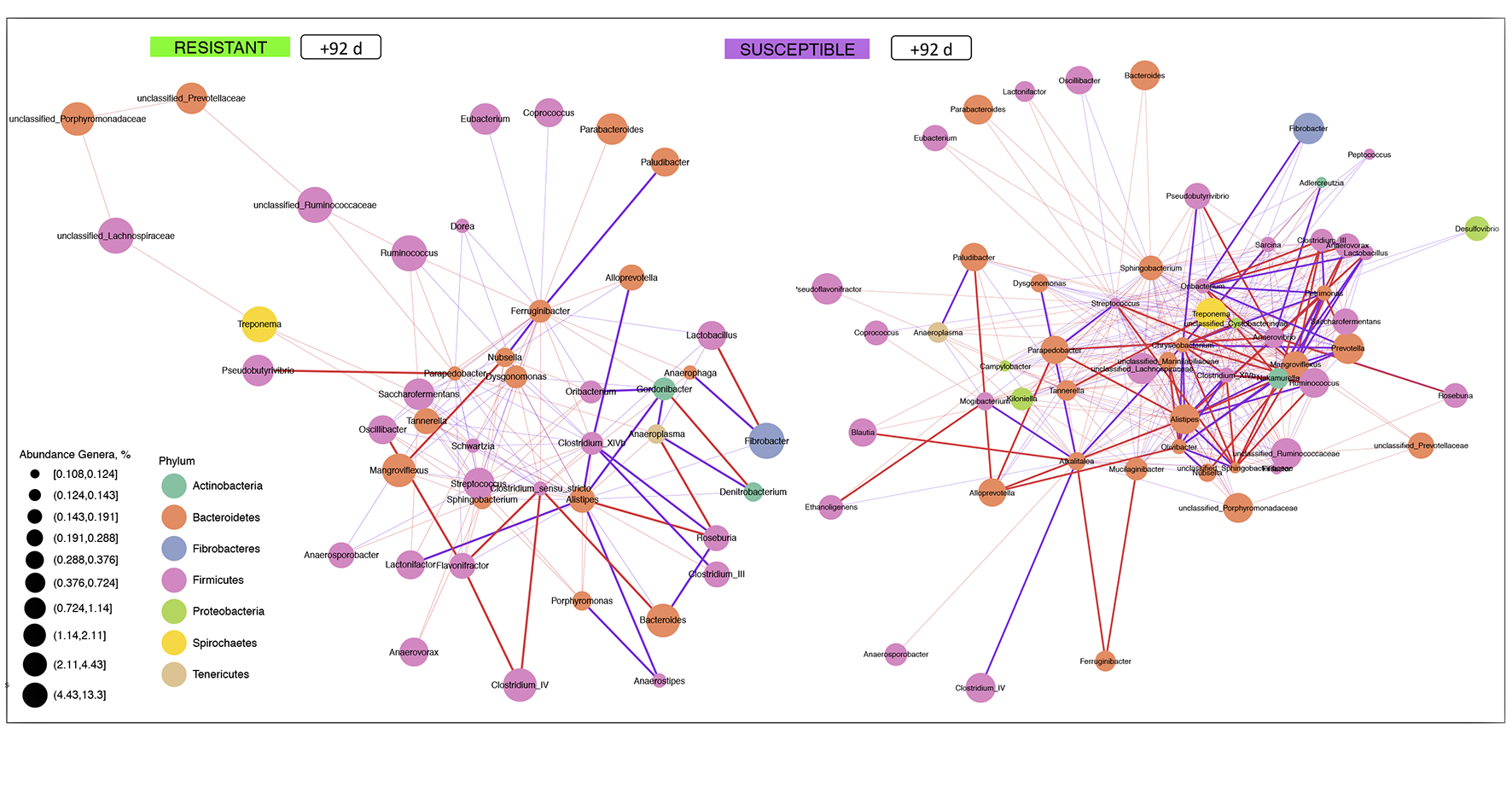

Supplement: Figure S10 — Co-occurrence genera network at 92 days after the entry to the pasture for susceptible and resistant ponies. The correlations among genera were calculated using the PCIT method, which identifies significant co-occurrence patterns. The size of the node is proportional to genera abundance. Node fill color corresponds to phylum taxonomic classification. Edges color represent positive (red) and negative (blue) connections, the edge thickness is equivalent to the correlation values. Only genera with a relative abundance >0.10 were included. [file Image10.TIF]
